# StrainGE: a toolkit to track and characterize low-abundance strains in complex microbial communities

**DOI:** 10.1186/s13059-022-02630-0

**Published:** 2022-03-07

**Authors:** Lucas R. van Dijk, Bruce J. Walker, Timothy J. Straub, Colin J. Worby, Alexandra Grote, Henry L. Schreiber, Christine Anyansi, Amy J. Pickering, Scott J. Hultgren, Abigail L. Manson, Thomas Abeel, Ashlee M. Earl

**Affiliations:** 1 Infectious Disease & Microbiome Program, Broad Institute, 415 Main Street, Cambridge, MA 02142 USA; 2 Delft Bioinformatics Lab, Delft University of Technology, Van Mourik Broekmanweg 6, Delft, 2628 XE The Netherlands; 3 Applied Invention, Cambridge, MA USA; 4 Department of Immunology and Infectious Diseases, Harvard T.H. Chan School of Public Health, Boston, MA 02115 USA; 5 Department of Molecular Microbiology, Washington University School of Medicine, St. Louis, MO 63110 USA; 6 Center for Women’s Infectious Disease Research (CWIDR), Washington University School of Medicine, St. Louis, MO 63110 USA; 7 Department of Civil and Environmental Engineering, University of California, Berkeley, Berkeley, CA 94720 USA; 8 Stuart B. Levy Center for Integrated Management of Antimicrobial Resistance (Levy CIMAR), Tufts University, Boston, MA USA

**Keywords:** Metagenomics, Microbiome, Strain-tracking

## Abstract

Human-associated microbial communities comprise not only complex mixtures of bacterial species, but also mixtures of conspecific strains, the implications of which are mostly unknown since strain level dynamics are underexplored due to the difficulties of studying them. We introduce the Strain Genome Explorer (StrainGE) toolkit, which deconvolves strain mixtures and characterizes component strains at the nucleotide level from short-read metagenomic sequencing with higher sensitivity and resolution than other tools. StrainGE is able to identify strains at 0.1x coverage and detect variants for multiple conspecific strains within a sample from coverages as low as 0.5x.

**Supplementary Information:**

The online version contains supplementary material available at 10.1186/s13059-022-02630-0.

## Background

Human-associated microbial communities include complex mixtures of bacterial species. Many of these species are renowned for their genomic and phenotypic plasticity. For example, strains of *Escherichia coli* share a core genome representing only about half of their genes [1] and cause distinct disease including diarrhea and urinary tract infections, or potentiate tumorigenesis, while other strains are able to co-exist with their host without causing overt illness [2–4]. Multiple distinct strains of the same species, often from genetically dissimilar phylogroups, frequently coexist within a single human gut community [5, 6], the implications of which are mostly underexplored due to the difficulties of studying strain-level variation from complex community samples.

While culture-based approaches have been a workhorse for dissecting strain-level diversity, these approaches can be slow and unfaithful to the true representation of strains, due to culturing bottlenecks that limit observed diversity, as well as the potential for evolution during culture [7]. Whole metagenome shotgun sequencing approaches offer less perturbed views of strain-level diversity, but require specialized computational tools. However, most current strain-level metagenomic data analytical tools (reviewed in Anyansi et al. [8]) were not designed to work at the low coverages typically found for many clinically relevant organisms in metagenomic samples, such as *E. coli* in the human gut [5]. Existing tools that aim to disentangle within-species strain mixtures include BIB [9], StrainEst [10], and DiTASiC [11], as well as the broader taxonomic profiling tools like Kraken2 [12] and GOTTCHA [13] when given an appropriate database. These tools rely upon a precomputed database of reference genomes, from which the best matches are reported for a sample (or set of samples). Thus, output from these tools is dependent upon database granularity and does not distinguish between distinct strains matching the same reference. Another class of tools characterizes and tracks strains based on single nucleotide variant (SNV) profiles along a single reference or a set of marker genes, including MIDAS [14], StrainPhlan [15], and ConStrains [16]. In the case of strain mixtures, MIDAS and StrainPhlan do not untangle the SNVs coming from different strains, while ConStrains attempts to link SNVs with similar allele frequencies, though linking SNVs requires high strain coverage to be accurate [16, 17]. A third class of tools aims to recover strain-level variation after de novo metagenomic assembly, including DESMAN [17], inStrain [18], and STRONG [19]. Assembly approaches require higher sequence coverage than typically achieved for lower abundance members of a community. To our knowledge, none of these computational approaches work robustly at low coverages (< 10x), accurately disentangle mixtures of same-species strains, and distinguish similar strains at the nucleotide level.

In order to be able to disentangle mixtures of low-abundance, clinically important strains within metagenomic data, we developed the Strain Genome Explorer (StrainGE) toolkit. In an advance over related tools, StrainGE works at exceptionally low sequence coverages (from 0.1x) to identify strains in a sample, and allows the user to characterize and compare strains across samples at the nucleotide level, with high resolution. We have extensively benchmarked StrainGE on synthetic data and compared it against other state-of-the-art strain detection tools. We also applied StrainGE to multiple clinical human gut metagenomic datasets, demonstrating StrainGE’s ability to glean insights into biological systems that previous tools could not, including observing previously undetected persistence of low-abundance strains across time. Herein, we applied StrainGE to analysis of clinically important strains of *E. coli* and *Enterococcus*, but StrainGE can be broadly applied to all community assemblages where same species bacterial strain dynamics are of interest.

## Results

### Strain Genome Explorer (StrainGE) toolkit

StrainGE is a toolkit for strain-level characterization and tracking of species (or genera) of interest from short read metagenomic datasets, tuned specifically to capture low abundance strains where data are scant. StrainGE has two key components: Strain Genome Search Tool (StrainGST) and Strain Genome Recovery (StrainGR). StrainGST sensitively reports reference genome(s) from a database that are most similar to the strain(s) in a sample. StrainGR analyzes short read alignments to a reported reference genome(s) to identify single nucleotide variants (SNVs) and large deletions (i.e., gaps in coverage) relative to the reference. Though StrainGST can be used as a standalone tool, the StrainGE tool suite, including StrainGR, enables sensitive nucleotide-level comparison and tracking of strains across multiple samples and provides insights into potential functional variation among individual strains.

**Fig. 1:**
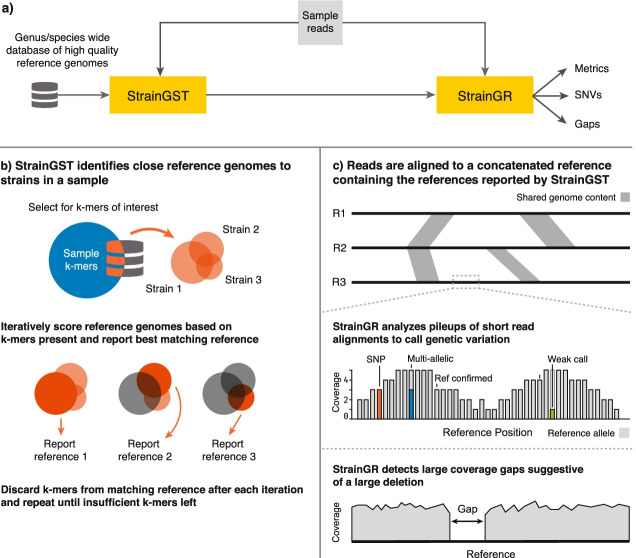
StrainGE is a toolkit to track, characterize and compare low-abundance strains in metagenomic samples. **a** Overview of StrainGE pipeline. StrainGST uses a database of high quality reference genomes to select those most similar to strains present in a metagenomic sample. StrainGR further characterizes SNVs and gaps that differ between references selected by StrainGST and the actual strain present in the sample. **b** At each iteration, StrainGST scores each reference strain by comparing the k-mer profile of the reference to the sample k-mers, reporting the reference closest to the highest abundant strain in the sample. The k-mers in the reported reference are removed from the sample and the process is repeated to search for lower-abundance strains, until there are insufficient k-mers. **c** StrainGR uses a short read alignment-based approach to characterize variation (SNVs and gaps) between the reference(s) identified by StrainGST and the metagenomic sample. Regions shared between the concatenated genomes (gray shaded areas) are detected and excluded from variant calling. Alleles are classified as “strong” or “weak.” After applying rigorous QC metrics, positions in the reference are classified as (i) “reference confirmed” (light gray; a single strong reference allele), (ii) “SNV” (red; a single strong alternative allele), or (iii) “multi-allelic” (blue; multiple strong alleles present, e.g. the blue allele together with the reference allele in gray). The position with a strong reference allele and a weak alternative allele (green; an allele with only limited support in the reads) is classified as “reference confirmed” because only the reference allele is considered strong at that position. The “callable” genome is defined as all positions within the reference with at least one strong allele call

In brief, StrainGST builds a database of high-quality reference genomes (e.g., RefSeq assemblies) from a species or genus of interest (Fig. 1a), filtering them to remove highly similar genomes using a k-mer based clustering approach, with a tunable threshold (Additional file 1: Table S1). StrainGST’s default database clustering threshold (0.9 Jaccard similarity) corresponds to an approximate ANI of ~ 99.8% [20], which determines the minimum distance between reference genomes. To identify a similar reference(s) to the strain(s) within a sample and to estimate its relative abundance, StrainGST compares the k-mers in the sample to those of the database reference genomes (Fig. 1b) and iteratively ranks each reference using three key metrics, similar to QuantTB [21]: (1) the fraction of reference k-mers present in the sample, (2) the fraction of sample k-mer counts explained by a reference, and (3) the evenness of the distribution of shared k-mers along a reference. If the resulting score is above a tunable threshold, the reference strain is reported as present in the sample.

StrainGR was designed to complement StrainGST by providing a more detailed view of the nucleotide- and gene-level differences between a strain in a sample and its closest reference, which can be used to compare across samples having strains that match the same reference. StrainGR analyzes alignments of metagenomic sequencing data to each StrainGST predicted reference (Fig. 1c). To ensure accurate SNV calls while maintaining sensitivity at low coverage, StrainGR employs stringent quality thresholds and heuristics to filter spurious alignments and reduce the number of incorrect calls.

To separate SNVs belonging to different strains, StrainGR creates a concatenated set of reference genomes, containing all references predicted by StrainGST in a sample or set of samples. It uses this reference set to align metagenomic reads and call variants. While close reference genome(s) generally result in more accurate alignments and variant calls [22], StrainGR still provides meaningful relationships when the reference is more distant, as would be the case in a smaller constructed database or with less well-studied organisms (Additional file 1: Supplemental Results A; Figs. S1-S4). To prevent assigning alleles incorrectly, StrainGR only calls variants in regions unique to a single reference by filtering out ambiguously aligned reads. In cases where StrainGST has identified distinct but closely related strains across samples, StrainGR can perform another, coarser round of reference clustering prior to concatenation in order to increase the amount of unique sequence for variant calling.

Variant calls can then be used to compare strains across samples. StrainGR compares positions within the “callable genome” or the set of positions with any reference or alternative allele supported by at least two good reads and > 10% of the alignment pileup (Fig. 1c). To perform a comparison, only “common callable” positions are considered, which represent the subset of the callable genome for a given reference that is shared by two samples. Strain relationships can be assessed using two key metrics: (i) the Average Callable Nucleotide Identity (ACNI), or the percentage of common callable positions where both samples have a single identical base call, and (ii) a “gap similarity” metric, as patterns of large deletions are often conserved between closely related strains, which can provide an orthogonal metric of strain similarity [23]. The ACNI and gap similarity values that define two samples as containing the same “strain” depend on the research question [7]. For the purposes of this manuscript, we consider two samples to contain the same strain if ACNI is ≥ 99.95%, which was based on our benchmarking of in silico *E. coli* spiked metagenomes. This threshold is stricter than our initial database clustering threshold of 99.8%, as samples matching the same reference in the database can contain different strains. As different studies may necessitate different strain definitions, we have intentionally made these thresholds easily tunable. With StrainGST able to accurately report close references to strains at coverages as low as 0.1x, and StrainGR able to track and characterize strains from 0.5x coverage, StrainGE enables sensitive analysis of very low-abundance strains, such as typical *E. coli* relative abundances of < 0.1% within a 3G metagenomic sample.

### Benchmarking StrainGE on *Escherichia*

StrainGE was designed to be broadly applicable across different bacterial genera and species, including less well-studied species lacking numerous high quality reference genomes (Additional file 1: Supplemental Results A). For benchmarking, we focused on *E. coli*, an evolutionarily and functionally diverse species. Despite their importance to human health, *E. coli* are typically found at low (< 1%) relative abundance in diverse strain mixtures in human guts [5].

We first used StrainGST to construct an *Escherichia*-specific reference database by downloading all available complete *Escherichia* assemblies from NCBI RefSeq (929 assemblies, July 2019; Materials and methods; Additional file 2). Because plasmids readily transfer between different genetic backgrounds of the same and/or different species (see Additional file 1: Supplementary Results A) [24], scaffolds labeled as plasmid, or those < 1 Mbp were removed. After using the default clustering threshold corresponding to ~ 99.8% ANI, the resulting StrainGST database contained 361 complete *Escherichia* chromosomes, comprising 341 *E. coli* and *Shigella* chromosomes representing all eight phylogroups [1], as well as 20 chromosomes from other *Escherichia* species.

#### StrainGE can accurately characterize strains and approximate ANI at coverages as low as 0.1x

To assess StrainGE’s ability to detect and characterize strains, we first benchmarked each of StrainGE’s components, StrainGST and StrainGR, individually. To benchmark StrainGST, we first used in silico constructed metagenomes that were spiked with sequences of known *Escherichia* strains at varying relative abundances. We compared StrainGST’s ability to identify the correct close reference to that of two similar tools that depend on reference databases, BIB [9] and StrainEst [10]. While the databases used for StrainGST and StrainEst were identical, BIB’s database construction method did not scale; thus, we used a smaller database with 20 genomes. StrainGST performed as well as, or better than, the other tools across all scenarios tested, including mixes of up to 3 strains at unequal abundances or 4 strains of equal abundance and stood out strongly when strains were at very low abundance (Additional file 1: Supplementary Results B; Fig. S5).

To further benchmark these three tools on real sequencing data of known strain composition, we created and sequenced a mock community containing approximately 99% human DNA and 1% *E. coli* DNA, representing a mixture of four distinct, previously sequenced strains with fully finished genomes mixed in unequal (approximately 80:15:4.9:0.1) relative abundances (Materials and methods). StrainGST resolved the composition of this in vitro mock community without error (Table 1), while other tools reported two or more false positives (Additional file 1: Table S2).

**Table 1:** StrainGST was the only tool that correctly identified the known composition of a mock community

Predicted strains (approximate ANI to closest true strain)^**a**^			
Actual strain in mock community (phylogroup)	StrainGST	StrainEst	BIB
*E. coli* SEC470 (A)	*✓*	*✓*	*E. coli* K-12 GM4792, 99.24 %
*E. coli* UTI89 (B2)	*E. coli* UM146, 99.95%	*E. coli* UM146, 99.95%	*E. coli* H105, 98.49%
*E. coli* Sakai (E)	*E. coli* 149, 99.89%	*E. coli* 149, 99.89%	*E. coli* 108, 99.97%
*E. coli* 24377A (B1)	*✓*	*✓*	*E. coli* S40, 99.01%
		✘ *E. coli* APEC IMT5155, 99.51%	✘ *S. flexneri* G1663, 97.97%
		✘ *E. coli* RM14721, 99.44%	✘ *E. coli* LHM10-1, 98.12%
			✘ *E. coli* MSHS 133, 97.67%
			✘ *S. dysenteriae* 80-547, 97.71%
			✘ *E. coli* IMT16316, 97.39%
			✘ *S. dysenteriae* ATCC 12039, 97.08%

^a^A check mark indicates that the exact strain was present in our database and correctly identified. A strain name indicates that the exact reference was not in the reference database, but the closest available reference was correctly identified (along with its approximate ANI to the actual strain). A strain name with an “X” indicates a false positive strain identified by the tool that was not present in the mock community. Percentages near strain names indicate approximate ANI to the closest true strain. Relative abundances for each strain are listed in Additional file 1: Table S2

**Fig. 2:**
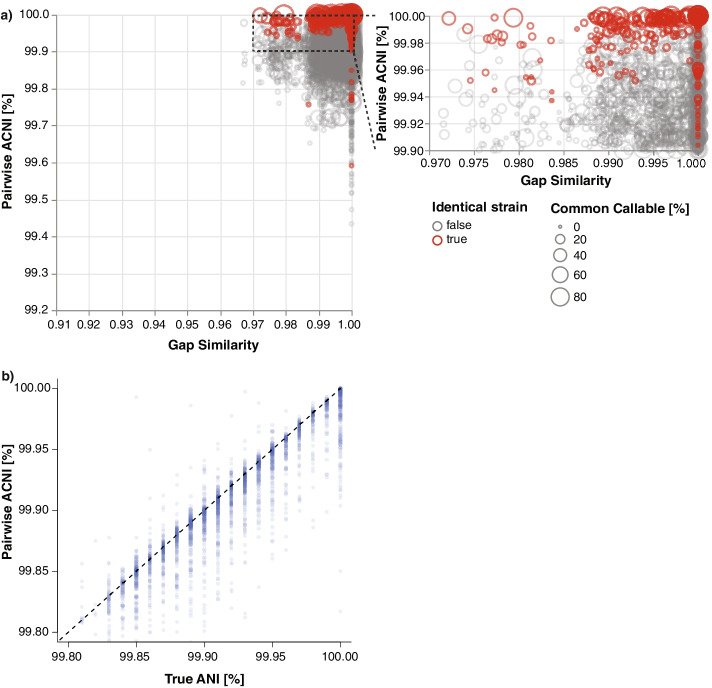
StrainGR discriminates between highly similar strains and reports ACNI which strongly correlates with true ANI. **a** For all synthetic sample pairs with the same StrainGST reference called, the Jaccard gap similarity index and pairwise ACNI are plotted. Circle size indicates the percentage of the reference genome that was callable across both strains being compared. Red circles indicate comparisons between identical strains. **b** For all pairs, the true ANI between spiked isolates is plotted against the ACNI, as estimated by StrainGR. The dashed line indicates parity between these metrics. Pairs of strains could have 0–10,000 SNV differences

To benchmark StrainGR’s ability to call variants (SNVs and large deletions, or gaps), we used another set of *Escherichia*-spiked metagenomes, with reads simulated from *in silico* mutated reference genomes (99.9% ANI to reference; 5,000 SNVs). StrainGR accurately called SNVs and large deletions, for both single strain and mixture samples, providing key information to assess whether two samples shared a strain via the ACNI metric, StrainGR’s approximation of ANI, and gap similarity (Additional file 1: Supplementary Results C-D; Fig. S6). To assess the accuracy and robustness of ACNI, we generated spiked metagenomes similar to those described above, but we varied the number of SNVs introduced in silico (100–99.9% ANI to reference; 0–5,000 SNVs) and used different metagenomic background samples, some with other *E. coli* strains present (Additional file 1: Table S3). Identical strains in different samples had high ACNI and gap similarity (Fig. 2a) and StrainGR’s ACNI across all strain pairs correlated strongly with true ANI, even though ACNI is based on unique regions, and ANI is based on the entire genome (Fig. 2b). In this benchmark, the optimal ACNI threshold to classify two samples as having the same strain was 99.98% (Additional file 1: Fig. S7), which is likely higher than the value we would expect from real data due to the artificially uniform distribution of SNVs in our synthetic benchmarks and that the references used in benchmarking were also present in the StrainGST database. For analysis of real data in this manuscript, we chose a slightly lower value of 99.95%.

#### StrainGE was the most accurate at detecting shared strains at coverages as low as 0.5x

**Fig. 3:**
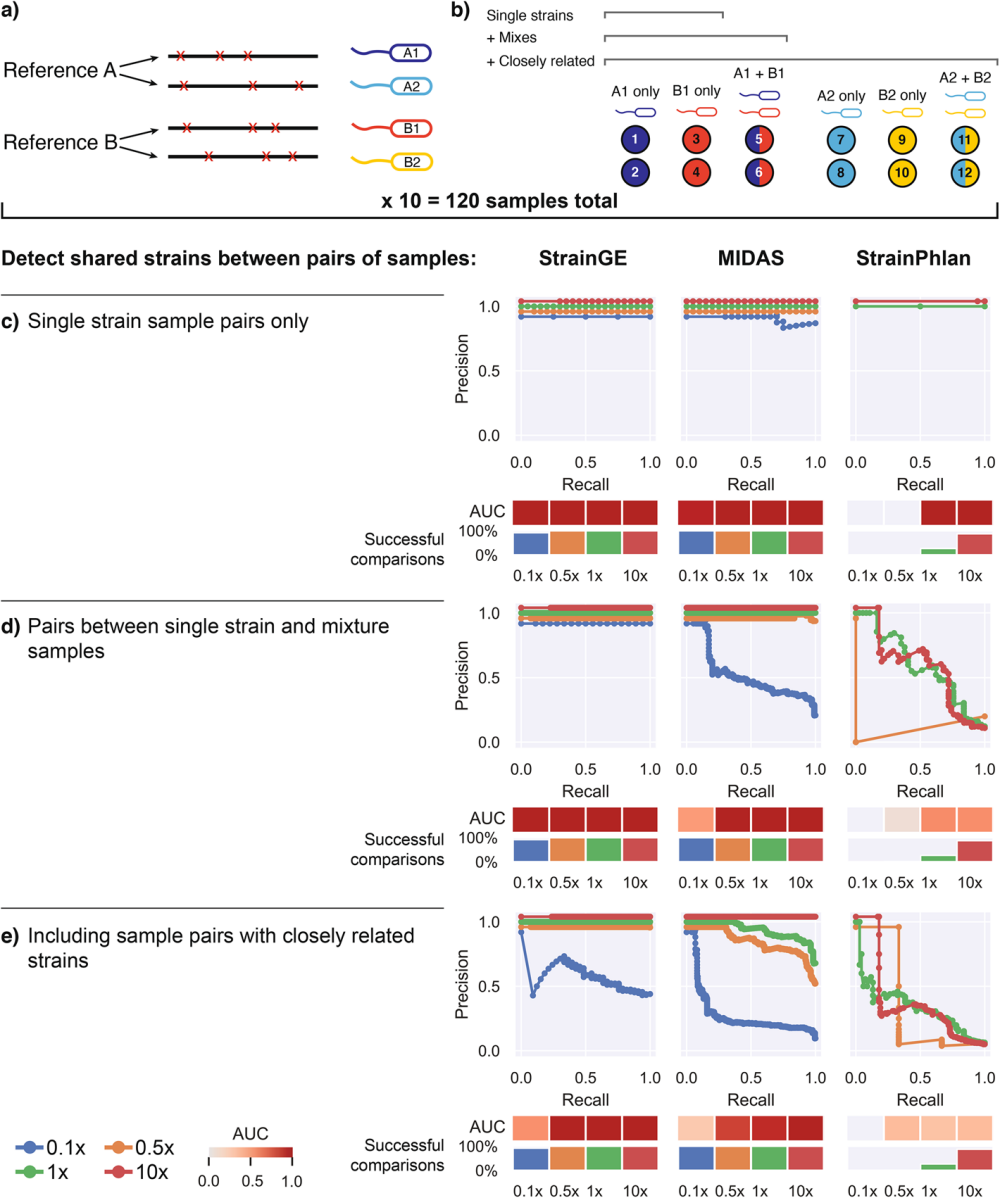
StrainGE is the only tool that can detect strain sharing at coverages as low as 0.5x. **a** Depiction of how synthetic *Escherichia* genomes were generated from randomly selected NCBI RefSeq genomes to create sets of closely related strains (e.g., A1/A2 and B1/B2) for spike in experiments. **b** Depiction of how spiked metagenomes were created using synthetic genomes from **a**. Each circle represents a spiked metagenome. The color of the circle indicates which synthetic strain was included: single color circles indicate spiked metagenomes containing a single synthetic strain, and two color circles indicate spiked metagenomes containing two synthetic strains mixed at equal proportions. **c**–**e** Precision-recall curves for each tool and coverage 0.1x–10x, when given the task to detect which sample pairs contain identical strains. The area under the curve (AUC) is depicted as a heatmap below. The “successful comparisons” bar plot indicates the percentage of sample pairs for which a comparison was possible (i.e., tools ran to completion for both samples). **c** Limiting to single-strain samples from distinct references. **d** Including samples with two strains, but limited to strains from distinct references. **e** Including samples with closely related strains

Having demonstrated that both StrainGST and StrainGR worked well, we aimed to assess StrainGE’s complete pipeline to track strains across samples, including in strain mixtures. We compared StrainGE’s ability to track strains to two recent, highly cited strain-tracking tools, MIDAS [14] and StrainPhlan [15]. Although MIDAS and StrainPhlan require high strain coverage to run to completion (5x and 10x, respectively), we were able to use manual tuning to allow these tools to accommodate our lower coverage benchmarks (Materials and methods). We excluded ConStrains [16] because of its high coverage requirements which could not easily be tuned [16, 17]. To assess the sensitivity of these tools to distinguish between similar strains, we generated pairs of spiked metagenomic samples, each containing one or more *Escherichia* strains at 0.1x-10x coverage. Similar strain pairs were derived from the same reference genome, but with a different set of ~ 5000 random SNVs introduced in silico into each strain’s genome (Fig. 3a, b). This resulted in each strain having 99.9% ANI to the reference and each strain pair having 99.8% ANI to one another. This identity level should result in strain pairs matching the same StrainGST reference but still distinguishable by StrainGR.

At 10x coverage, MIDAS and StrainPhlan performed comparably using tuned (Fig. 3) and default (Additional file 1: Fig. S8) settings. While StrainGE and MIDAS performed well across all scenarios at high coverage, StrainPhlan performed poorly on mixes because it only reported a single SNV profile for each sample. For lower coverage scenarios, StrainGE consistently outperformed the manually tuned versions of MIDAS and StrainPhlan (Fig. 3). For single strain samples, StrainGE perfectly matched strain pairs down to 0.1x coverage, with MIDAS performing comparably (Fig. 3c). StrainPhlan performed marginally at 1x coverage and still was unable to run to completion at coverages lower than 1x. For simple mixtures (Fig. 3d), only StrainGE and MIDAS correctly matched most pairs, because StrainPhlan was unable to disentangle mixes. StrainGE was the only tool that was able to generate results across the whole range of coverages, scoring almost perfectly down to the lowest tested coverage of 0.1x. When we included samples containing very closely related pairs (Fig. 3e), StrainGE and MIDAS performed well down to 0.5x coverage, but StrainPhlan could not distinguish between closely related strains, even at 10x coverage, likely due to its reliance on marker genes which comprise only a small fraction of the genome. Whereas StrainGE relied on a mean callable genome of 74% ± 13% (at 10x coverage), StrainPhlan relied on marker genes which only covered on average 1.4% ± 0.3% of the references.

StrainGE achieved this high sensitivity with comparable runtime to MIDAS and StrainPhlan, and its memory usage was well within the range of modern cluster systems or powerful personal computers (Additional file 1: Fig. S9). Another key advantage of StrainGE over the other tools is its ability to link a strain in a sample to its specific close reference genome reported by StrainGST, which places an observed strain within the known phylogenetic structure of the reference database (Additional file 1: Fig. S10). In contrast, the SNV profiles outputted by StrainPhlan (based on marker genes) or MIDAS (compared to a single built in *E. coli* reference) do not offer convenient phylogenetic placement.

### In real metagenomic data, StrainGE identifies low abundance strains and can track strains across samples, including in strain mixtures

#### StrainGE can identify lower abundance instances of persistent strains previously undetectable by other tools

In order to assess StrainGE’s utility to characterize strains from real world samples, we examined its performance, using default parameters with our *Escherichia* reference database, on a previously published metagenomic dataset of 27 longitudinally collected stool samples from a patient with Crohn’s disease, upon which MIDAS was run to delineate *E. coli* strains [25]. MIDAS identified seven dominant “strain types” (“ST1” – “ST7”) that varied in abundance over time. Each of these belonged to a distinct multi-locus sequence type (MLST) and represented one of five *E. coli* phylogroups. StrainGE showed good concordance with results from MIDAS for all high abundance strains (> 10% abundance) (Table 2). For the two calls that disagreed, our StrainGST database lacked representatives for the two MLSTs reported by MIDAS. However, StrainGE selected the next closest reference, which we confirmed by comparing the whole genome sequence from a cultured representative of ST1 [25] to our reference database.

**Table 2:** The strains predicted by MIDAS match the dominant strains predicted by StrainGE

Strain (time points)	MIDAS^**a**^		StrainGST		
	MLST	***E. coli*** phylogroup	MLST	***E. coli*** phylogroup	Most abundant strain
**ST1** (1)	95	B2	95	B2	PA45B^b^
**ST2** (2)	1629^c^	E	1011	E	Santai
**ST3** (3,4)	69	D	69	D	118UI
**ST4** (11-18)	58	B1	58	B1	D5
**ST5** (19-22, 27)	131	B2	131	B2	MVAST0167
**ST6** (23,24)	409^c^	A	1408	A	AR_0061
**ST7** (25,26)	1727	B1	1727	B1	2011C-3911

^a^Fang et al. [25]

^b^The actual strain corresponding to ST1 (3_2_53FAA) was whole-genome sequenced by Fang et al. PA45B and 3_2_53FAA share 99.9% average nucleotide identity based on whole-genome comparative genomics analysis

^c^MLST profile was not represented by any reference genome in the StrainGE database. StrainGST predicted the closest reference within the StrainGE database, which was within the same phylogroup

**Fig. 4:**
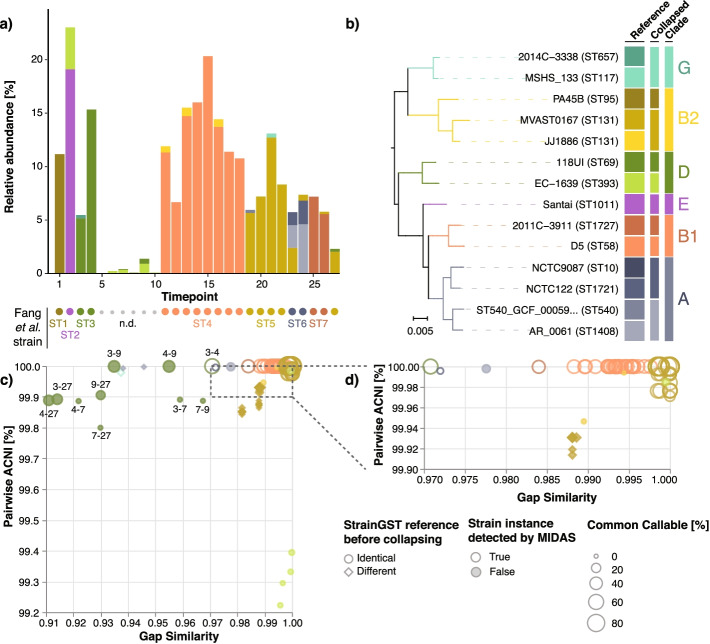
StrainGE identified previously undetected low-abundance strains in longitudinal samples from an individual with Crohn’s disease. **a** Stacked barplot showing the relative abundances of StrainGST calls for each of 27 longitudinal stool metagenomes from Fang et al [25]. Circles indicate the strain detected in Fang et al., colored by its StrainGST counterpart and labeled using the ST designations (ST1-ST7) assigned by Fang et al. Small gray circles indicate samples where no strain was predicted in Fang et al.; these are labeled with “n.d.” **b** Single-copy core phylogeny of the 14 StrainGST reference genomes with close matches to strains across samples. Colors are based on the reference’s clade; see column “Clade”. “Collapsed” column indicates which reference was selected as a representative for subsequent StrainGR analysis, when two or more references shared more than ~ 99.2% ANI. **c** For all sample pairs matching the same collapsed reference, the Jaccard gap similarity index and pairwise ACNI are plotted. Circles indicate comparisons where the predicted reference was the same before collapsing, and diamonds indicate cases where the predicted reference before collapsing was different. Sizes of shapes indicate the percentage of the reference genome that was callable across both strains being compared. Filled in shapes indicate whether this strain instance was undetected by MIDAS. Dark green circles are labeled with the time points compared. **d** Zoomed in view of the upper right corner of **c**)

StrainGST also identified seven distinct strains that were missed by MIDAS (Fig. 4a). While the majority of these were secondary strains found to coexist with a dominant strain predicted by MIDAS, StrainGST also predicted strains at time points where MIDAS called none (time points 6–10). In most of these cases, the strains were at ≤ 1% relative abundance and were also detected by MIDAS at higher abundance in other time points (e.g., ST3; dark green), lending credence to their existence in these samples and suggesting that some strains were more persistent over time than previously predicted (Fig. 4a).

We ran StrainGR on all datasets using a concatenated reference including 10 out of 14 total references reported by StrainGST to ensure each genome had at least 20% unique genome content (Materials and methods; Fig. 4b). SNV and gap patterns predicted by StrainGR showed that the majority of strains matching the same reference had strikingly high pairwise ACNI (> 99.96%) and gap similarity (> 0.97) (Fig. 4c, d), which were within the range of those of same-strain sample pairs in our simulations (Fig. 2a). However, StrainGR results from strains matching the *E. coli* 118UI (dark green) reference stood out. While 118UI-like strains from samples 3 and 4 had ACNI and gap similarity relationships that were on par with what we observed in same-strain simulations, all other comparisons fell outside of this range, suggesting that this individual carried a mixture of 118UI-like strains in their gut over time that were closely related, but not necessarily the same with respect to gene content and single nucleotide variation (Additional file 1: Fig. S11).

#### StrainGE accurately and sensitively identified a low-abundance, persistent strain of *E. coli* in longitudinal stool samples from a woman with recurrent urinary tract infection

Although the results of StrainGE on the Fang et al. [25] dataset highlighted its ability to resolve strains present at low abundance, the overall *E. coli* relative abundances in these samples were significantly higher (median 7.9%; range 0.05–27%) than those typically seen in the human gut. Thus, we also tested StrainGE on 12 stool metagenomes having more typical *E. coli* relative abundances (median 0.55%; range 0.006–17.4%), which originated from a single individual with a history of recurrent urinary tract infection (rUTI) over the span of a year. Given that the gut is a known important reservoir for UTI-causing *E. coli* [26], it was of interest to trace gut *E. coli* strain dynamics and their relationship with UTI.

**Fig. 5:**
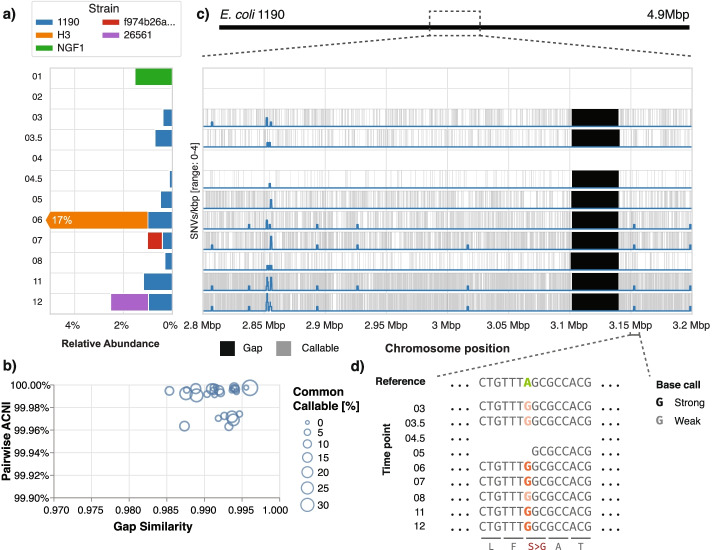
StrainGE detected a long-term, persistent strain of *E. coli* in a woman with rUTI. **a** Relative abundances predicted by StrainGE are shown for all *E. coli* strains detected. **b** For all sample pairs containing a strain matching to *E. coli* 1190, plot shows pairwise ACNI and gap similarity scores. Size of the circle indicates the percentage of the common callable genome. **c** Zoom in on a region of the chromosome of *E. coli* 1190. Gray shaded areas indicate “callable” regions, where StrainGR had enough read data to make a strong allele call. Predicted gaps are shaded black. The blue line represents the number of SNVs per 1,000 bp, observed in at least 3 samples. **d** Further zoom-in representing a region where StrainGR identified a nonsynonymous SNV that was consistently detected across all 1190-like strains

StrainGST detected a total of five distinct strains of *E. coli* (Fig. 5a), including a recurrent strain detected in over half of samples. The persistent strain, an *E. coli* 1190-like strain from phylogroup D, had a median relative abundance of only 0.6% (range 0–1.2%) and was detected even in samples that were composed of multiple *E. coli* strains, including at very low (20-fold less) abundance relative to another strain (Fig. 5a). Despite its low abundance, we were able to confirm that all *E. coli* 1190-like strains had extremely high ACNI (> 99.95%) and gap similarity (> 0.98) (Fig. 5b), in line with the identities observed for same-strain benchmarking (Fig. 3a), suggesting that this strain, also the causative agent of this individual’s rUTI, persisted long-term in their gut.

Further, StrainGR output enabled us to look closely at the locations and identities of SNVs and genes within gaps relative to the reference. For example, we consistently identified a large gap across all time points encoding a prophage found in the original reference, but apparently lacking in the *E. coli* 1190-like strain in this individual (Fig. 5c). Using StrainGR output that included both strong and weak variant calls (see Fig. 1c for strong vs. weak calls; Materials and methods), we were able to track 839 variant sites across samples, where the corresponding allele was strongly called in at least one sample, and weakly called in at least five samples (e.g., a nonsynonymous SNV in the gene *cydC*; Fig. 5d). At each of the 839 variant sites, the called allele was identical across all time points, except for three sites where another secondary weak allele was called, further supporting the persistence of a single UTI-causing strain.

#### StrainGE accurately recapitulated known strain-level diversity from metagenomes and traced strains from mother to child

To demonstrate StrainGE’s applicability to other bacterial genera, we selected a previously published dataset investigating the impact of mode of delivery on the infant gut microbiome, including transmission and carriage of opportunistic pathogens from the *Enterococcus* genus [27]. Shao et al. longitudinally followed 596 babies (and 175 mothers) by collecting stool samples that were then whole metagenome shotgun sequenced and cultured for pathogens, including 451 enterococci that were then whole genome sequenced. This dataset allowed us to evaluate StrainGE’s ability to report on (i) the relationships between enterococcal strains predicted directly from metagenomes in comparison to those calculated from the genomes of cultured isolates and (ii) mother and child strain sharing. Furthermore, this dataset allowed us to evaluate StrainGE’s ability to predict and compare strains across samples using a sparser database, as there were fewer than a third as many RefSeq complete *Enterococcus* genomes than for *Escherichia*.

**Fig. 6:**
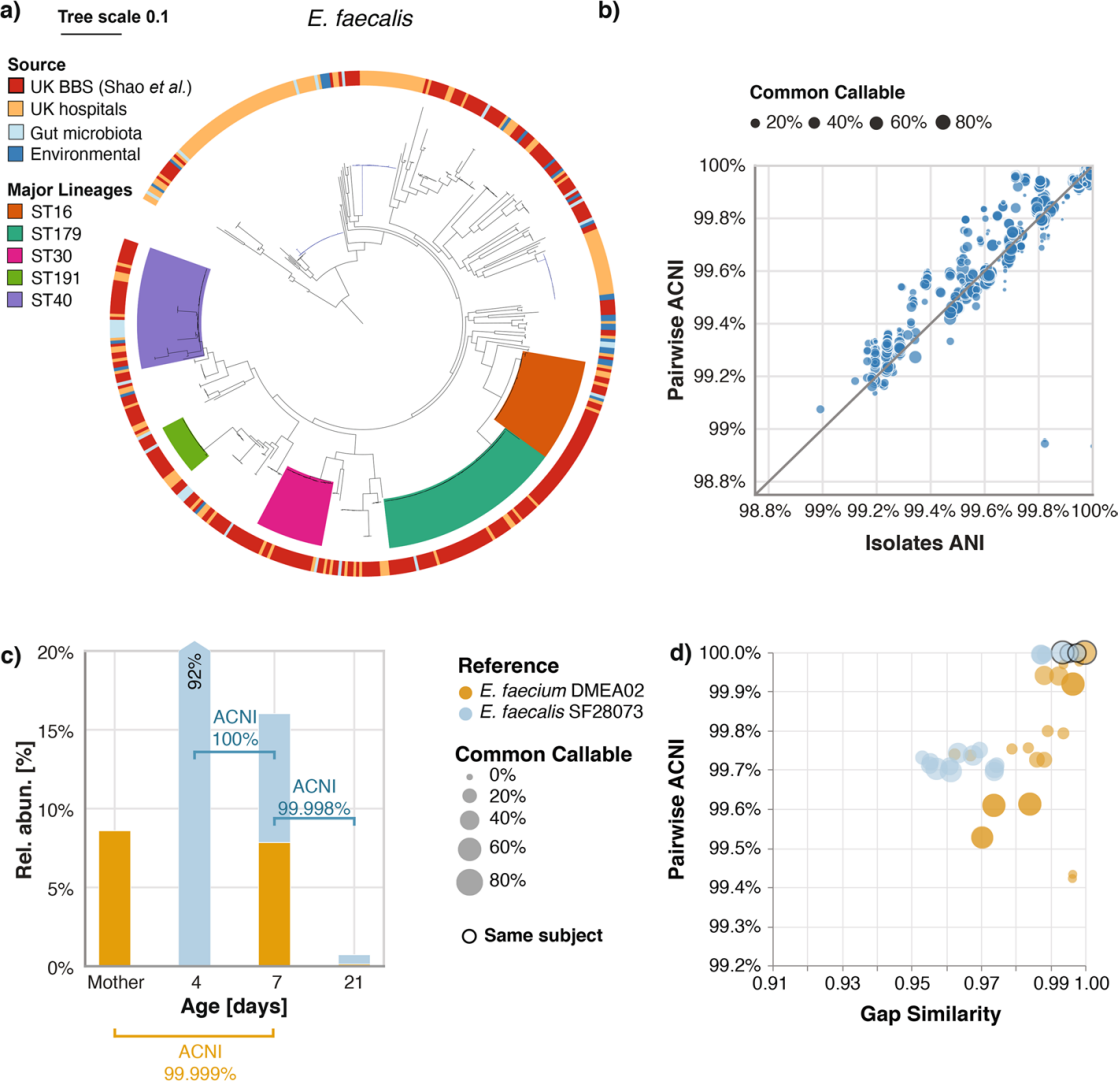
StrainGE recapitulates strain-diversity among bacterial isolates using metagenomic data only. **a** Single-copy core phylogenetic tree of *E. faecalis* isolates from the UK Baby Biome Study (UK BBS) (*n* = 282) in the context of isolates from other public UK hospitals (*n* = 168), human gut microbiota (*n =* 28), or other environmental sources (*n =* 27). Five major lineages were identified, represented by ST16, ST179, ST30, ST191, and ST40. Tree republished with permission from Shao et al. [27]. **b** Scatterplot relating ANI between isolates (x-axis) to StrainGE’s computed ACNI between metagenomes from which the isolates were derived (*y*-axis). **c** Barplot showing StrainGST predicted references and their relative abundances (*y*-axis) for strains present in metagenomic samples from a mother and her child taken over several days (*x*-axis). Strains matching the same reference are shown in the same color. Lines connecting bars are labeled with StrainGR computed ACNI. **d** For all pairs of samples with a strain close to either *E. faecium* DMEA02 (yellow) or *E. faecalis* SF28073 (blue), ACNI (*y*-axis) and gap similarity are plotted (*x*-axis). Circles with a black border represent pairs of samples from the same subject (or its mother). Size of the circle represents the percentage of common callable genome

We built a 163-member StrainGST database representing references from 80 *E. faecium*, 39 *E. faecalis* and 44 other enterococcal species (Materials and methods; Additional file 2) and ran StrainGE on all 1,679 stool metagenomes. StrainGE identified strain relationships that were very similar to those Shao et al. obtained using bacterial isolate comparisons. For example, the species distributions were roughly similar (Additional file 1: Table S4) and nearly half (42%) of references predicted by StrainGST belonged to one of the five major *E. faecalis* lineages previously identified (Fig. 6a). The pairwise ACNI distributions for strains matching these references mirrored the tree topology (Additional file 1: Fig. S12), and across the whole data set pairwise ACNI correlated strongly with ANI between corresponding isolates (Pearson’s *r* = 0.96; Fig. 6b; Materials and methods).

Shao et al. used StrainPhlan [15] to predict instances of mother-to-child strain sharing, including 7 *E. faecalis* and 2 *E. faecium* transmission events. Though no direct comparison of transmission predictions could be made (sample names were not reported), we hypothesize that StrainGE’s predictions would be more accurate since StrainPhlan’s marker genes covered only 3.6% ± 1.7% of reported *Enterococcus* genomes, while StrainGE’s callable genome was on average 39% ± 33%. Using StrainGE, we identified 17 mother-baby pairs for which StrainGST reported the same reference, of which six had sufficient common callable genome to calculate ACNI. Three pairs had ACNI < 99.7% and three had ACNI near 100%, including an example at 99.999% (Fig. 6c) suggesting that there were at least three instances of mother-baby strain sharing that we could confidently call based on our “same strain” ACNI threshold of 99.95%. Comparisons of strains matching the same reference from other mothers or babies revealed that they generally had considerably lower ACNI and gap similarity (Fig. 6d).

## Discussion

The ability to discern strain-level variation from primary specimens—where the species of interest may be at low abundance—can transform our understanding of species populations, ecologies, and transmission patterns. We have shown that our novel tool suite, StrainGE, is easy to use for ultra-sensitive detection of strains in primary specimen metagenomes. StrainGE uses both k-mer and alignment analysis to characterize sample strain genomes, including their (i) closest matching reference, which places them phylogenetically, (ii) relative abundance, and (iii) estimated ANI (ACNI) to other strains, which can be achieved even at very low coverage levels, with more detailed information about specific variants and cross-sample comparisons becoming available as coverage increases. StrainGE can provide nucleotide level resolution for individual bacterial strains or strain mixes that are present at 0.1% relative abundance, e.g., 0.5x coverage for a 5 Mb genome within 3Gb of sequencing reads. StrainGE provides a substantial advance over previously published tools, which (i) were not designed to work at these low coverages [16], (ii) report only overall consensus SNV profiles for a mixture [14, 15], or (iii) do not offer nucleotide-level resolution [9–11].

In addition to demonstrating good performance on an extensive array of benchmarking samples, we showed that StrainGE provided insights into the strain-level dynamics of bacteria in three real-world sample sets. For a patient with Crohn’s disease, StrainGE identified co-existing strains and strains at time points missed by another popular strain-tracking tool. StrainGE similarly was used to identify the long-time gut carriage of a low abundant UTI-causing *E. coli* strain, which we could track via stereotypical gene absence and SNP patterns, reported by StrainGE, that could be discerned even when other strains were present. Finally, using metagenomic data from primary stool specimens, StrainGE was able to recapitulate relationships among *E. faecalis* previously observed using whole genome sequencing of isolates and phylogenetic reconstruction, as well as provide strong evidence for transmission of *E. faecium* strains from mothers to their children. For this vignette, we used an ACNI threshold that we empirically determined to represent the same strain from in silico experiments. However, the measures that define “same” versus “different” strains will depend upon the research question and the species being evaluated [7]. StrainGE provides a compendium of outputs for assessing relationships between strains in detail, which can be used to evaluate appropriate thresholds for any system.

While we demonstrated StrainGE on a narrow set of bacterial species, StrainGE is designed to be broadly applicable to any genus or species, with a wide range of database sizes. While a dense database is generally preferred because the accuracy of variant calls improves with genetically closer references [22], our benchmarking showcased that StrainGST and StrainGR combined can return accurate information about strain relationships, even when few reference genomes are available. Furthermore, the default database clustering threshold of 99.8% is tunable to adjust for the number of references StrainGST considers since, for example, a very dense database could cause StrainGST to report different, but closely related references for two samples containing the same strain. To balance these two factors, we included a tool “prepare-ref” in the StrainGE suite, which performs an additional coarser round of clustering of StrainGST-determined references for a set of samples in order to select a smaller set of representatives prior to running StrainGR. This step increases the total amount of unique content across references to be considered in ACNI calculations and enables direct comparisons of more strains with respect to their nucleotide and gap similarities.

While StrainGST and StrainGR were designed to work together, both tools can work in isolation and provide useful standalone output. StrainGST with a dense database can provide fast phylogenetic placement of strains. Though not shown here, this also works on whole genome sequence data from bacterial isolates, providing a quick snapshot of phylogenetic relationships without needing to perform reference alignments or other more time-consuming phylogenomic pipelines. StrainGR could be used without StrainGST when good quality assemblies are available for strains present within a mixed community dataset. For example, long read sequencing and assembly of isolates or even whole metagenomes from a select number of time points could provide high quality substrates for StrainGR evaluations of strains in short read time series data.

Though competitive and filling a niche left behind by other strain-tracking tools, StrainGE has several limitations. It evaluates the relationships between strains using only unique regions of reference genomes, is unable to detect new genes that occur in strain genomes that are not present in its closest matching reference, and currently only works with Illumina data. Furthermore, StrainGE is currently not designed to phase SNVs from multiple strains matching the same reference in the same sample. In this case, StrainGR will output evidence for multiple alleles, but the frequencies of which cannot be robustly compared to link alleles together at the coverages under which StrainGE was designed to operate.

## Conclusions

Here, we present StrainGE, a novel suite of tools to characterize conspecific strains in complex microbial communities. We have demonstrated its accuracy using benchmarks and have shown that it represents a major advance over other published tools. Using three clinical metagenomic time series, we demonstrated its ability to yield insights into biological systems that previous tools could not, including the persistence of low-abundance strains across time. StrainGE’s sensitivity at very low coverages (0.1x and higher) will help to accelerate our understanding of the role of strain-level variation in shaping ecological and disease processes.

StrainGE is installable through bioconda and available at https://github.com/broadinstitute/strainge.

## Materials and methods

### Strain Genome Explorer toolkit algorithms

#### StrainGST: Strain Genome Search Tool

StrainGST is a k-mer based tool used to identify specific strain(s) of a species in a metagenomic sample. StrainGST computes a reference database of previously sequenced strains from this species and uses it to report close reference genomes to strains present in a metagenomic sample along with their relative abundances. The references reported by StrainGST can be used as input to StrainGR to further characterize genetic variation found within the metagenomic sample.

##### Creating a StrainGST database

A StrainGST database is constructed from a set of high quality sequenced reference genomes for a single species or genus, such as all complete reference genomes in NCBI RefSeq. From this set of genomes, StrainGST generates a database of k-mer profiles, using a sliding window (window size k) to traverse each genome and count the frequency of each k-mer. To reduce memory usage and computation time, a minhash technique (similarly to Mash [20]) is applied to keep 1% of the k-mers with the lowest hashes.

StrainGST next performs clustering to remove highly similar genomes from the reference set. In order to track and compare genomic variation across related samples, StrainGR must be able to align reads to a common reference genome across different sample sets. Therefore, the references reported by StrainGST should not be too closely related, or each sample could end up matching distinct yet closely related references, making comparisons difficult. StrainGST computes pairwise Jaccard similarities using each reference genome’s k-mer set, performing single linkage clustering using a Jaccard similarity threshold of *τ*, and picking a single representative genome for each cluster to include in the reference set. StrainGST selects the genome with the highest mean similarity to all other genomes in that cluster. This process ensures that the k-mer similarities between remaining genomes in the database are all lower than *τ*. Additionally, to remove genomes from the database that are highly similar to another genome, but that may have lower Jaccard similarity due to the presence of large indels, StrainGST removes genomes where 99% or more k-mers overlapped with those from another genome.

##### Identifying strains present in a sample

StrainGST uses this database to identify the closest reference genome(s) to the strain(s) present within a sample (Fig. 1). First, all reads in the sample are k-merized, resulting in the k-mer set *K*_*sample*_. The algorithm then selects for k-mers from the species of interest by taking the intersection between the sample k-mer set and that of the reference database for the species of interest (Fig. 1b), excluding k-mers not associated with the target species.

StrainGST then uses these k-mers to identify the reference genome(s) with the best k-mer matches to the sample using an iterative process. In each iteration, StrainGST scores each reference genome in the database against the remaining k-mers in *K*_*sample*_ in order to find the reference with the best score, which is reported to the user as the reference with the strongest evidence of being present. The scoring system is described in detail below. If no reference strain is identified that scores above a threshold *θ* (adjustable by a command line option), the algorithm is terminated. The default value for *θ* (0.02) was optimized to maximize sensitivity while minimizing false positives. In each iteration, k-mers corresponding to the reference selected are removed from the sample k-mer set in order to enable identification of secondary strains in the next iteration. This process continues until either no strain is reported or the maximum number of iterations is reached (default of 5).

##### Scoring metric for selecting matching reference strains

To determine which reference strain to report in each iteration, we calculate a score for each reference strain using a combination of three metrics based on (1) the fraction of matching k-mers in the reference, (2) the fraction sample k-mer counts that could be explained by this reference genome, and (3) the evenness of the distribution of matching k-mers across the genome.

1. Fraction of matching k-mers in the reference (*ƒ*)

This metric represents the fraction of distinct k-mers in reference *j* that is present in the sample and has a value between 0 and 1, where 1 would indicate all k-mers of this reference are present in this sample.

$${\begin{array}{c}{K}^{\prime }={K}_{sample}\cap {K}_j,\\ {}f=\frac{\mid {K}^{\prime}\mid }{\mid {K}_j\mid }.\end{array}}$$

*K*_*sample*_ represents all k-mers in the sample, *K*_*j*_ represents all k-mers in reference *j*, and *K*^′^ represents the set of k-mers both present in the reference and in the sample.

2) Fraction of sample k-mer counts that could be explained by this reference (*ɑ*)

To give more weight to reference genomes that are similar to higher abundance strains in the sample, StrainGST calculates the fraction of database k-mers remaining in the sample that could be explained by the k-mers in this reference:

$$a=\frac{\sum_{i\in K\prime }{c}_i}{\sum_{i\in {K}_{sample}}{c}_i}.$$

*c*_*i*_ represents the count of k-mer *i* in the sample. Note that we include k-mer counts, rather than using the fraction of *distinct* k-mers, which gives more weight to reference strains with high average depth of coverage. This metric has a value between 0 and 1.

3) Evenness (*e*)

To quantify whether the matching k-mers are evenly distributed across the reference genome, rather than being found predominantly in a small region (e.g., due to a horizontal gene transfer event, or conserved regions attracting reads from different species), we defined the *evenness* score. First, we assumed that the coverage across the genome follows a Poisson distribution. The rate parameter *λ*_*j*_ of the Poisson distribution specifies the average depth of coverage across the whole genome:

$${\lambda}_j=\frac{1}{\mid {K}_j\mid}\sum_{i\in {K}_j}\frac{c_i}{d_{ij}}$$

Here, *c*_*i*_ represents the count of k-mer *i* in the sample, and *d*_*ij*_ represents the count of k-mer *i* in reference strain *j*.

If *X* is the random variable that indicates how many reads cover a position, then using the Poisson distribution, the probability of observing *x* reads at a position is:

$$P\left(X=x\right)=\frac{\lambda_j^x\exp \left(-{\lambda}_j\right)}{x!}$$

The probability of observing 0 reads at a position is then *P*(*X* = 0) = exp(−*λ*_*j*_). The probability of observing at least one read at a position is [28]:

$$P\left(X>0\right)=1-P\left(X=0\right)=1-\exp \left(-{\lambda}_j\right)$$

This probability also represents the expected fraction of the genome covered by at least one read given a certain average depth of coverage. The evenness score describes how well the observed fraction of the genome covered by at least one read (which is estimated using the fraction of matching k-mers in the reference defined earlier) matches the expected fraction of the genome covered by at least one read when assuming a Poisson distribution for the depth of coverage:

$$e=\frac{f}{1-\exp \left(-{\lambda}_j\right)}$$

This score will be close to 1 if the observed fraction of the genome with at least one read matches the expected value for a certain average depth of coverage (assuming a Poisson distribution). It will be closer to zero if only small portions of the genome are well covered. A value higher than 1 indicates that the observed fraction of the genome with at least one read is higher than the expected fraction of the genome with at least one read. To bound this score between 0 and 1, StrainGST uses the minimum of *e* and its reciprocal:

$${e}^{\prime }=\min \left(e,\frac{1}{e}\right)$$

Finally, we combined these three metrics together in order to obtain the final score:

$$score=f\cdot a\cdot {e}^{\prime 2}$$

At each iteration, the reference strain with the highest score represents the best match to the highest abundant strain remaining in the sample and is reported to the user.

#### StrainGR: Strain Genome Recovery

The StrainGR pipeline consists of (1) building a concatenated reference based on reference strains reported by StrainGST, (2) aligning reads to the concatenated reference, (3) analyzing read alignments to call SNVs and large deletions, and (4) using these variant calls to analyze gene content or track strains across multiple samples.

##### Preparing a concatenated reference

To analyze a set of related samples together, such as a longitudinal series, StrainGR concatenates a single, unified set of representative references present across the whole dataset. This can facilitate comparisons of alignments or genomic variation across a set of samples, which may contain different strain mixes at different time points. Use of the concatenated reference allows reads with an allele unique to a particular strain to be aligned to the genome of the correct reference strain, thus helping disentangle reads from mixture samples. Genomes from the same species, however, will share conserved genomic regions (*i.e.*, housekeeping and other core genes), where the aligner will be unable to place reads unambiguously within the concatenated reference. StrainGR detects and excludes these conserved regions from variant calling.

In order to minimize conserved regions where StrainGR is unable to call variants, it is important to select a set of reference strains that are not too closely related, which could result in a large fraction of the concatenated reference genome being marked as shared. To construct a concatenated set of references with an optimal degree of similarity, StrainGR includes a tool called prepare-ref that analyzes StrainGST output from a set of samples (e.g., a longitudinal set from a single patient) and generates a concatenated reference ready for use with StrainGR, optionally performing another round of clustering at a stricter threshold to prevent too-closely related genomes from being included. By default, the stricter clustering threshold is set to a Jaccard similarity of 0.7 (~ 99.2% estimated ANI).

##### Read alignment and filtering

The reads from a metagenomic sample are then aligned to the concatenated reference using BWA-MEM [29], removing read pairs with (1) improper pairing, (2) clipped alignment, or (3) implied insert size smaller than the read length. In order to identify shared regions within the concatenated reference which should be excluded from variant calling, StrainGR tracks the number of “multi-mappable” read alignments (those which map equally well at multiple locations) at each position in the reference. When the majority of aligned reads at a position are multi-mappable, StrainGR excludes this position from variant calling. We rely on BWA’s “XA” SAM tag to obtain a read’s alternative alignment locations, so aligners other than BWA are not currently supported by StrainGR.

In addition to excluding multi-mappable regions, StrainGR also excludes regions with abnormally high coverage (greater than threshold *τ*), likely due to genes highly conserved across genera which attract nonspecific reads from other members of the microbial community. *τ* was chosen such that the probability of observing a depth of coverage higher than *τ* is 1 × 10^−7^ assuming a Poisson distribution. This value results in a threshold of 10x coverage when the mean coverage depth across the genome is 1x, and a threshold of 20x when the mean is 5x.

##### SNV calling

StrainGR analyzes read alignments to identify single-nucleotide variants (SNVs) between a specific strain within a metagenomic sample and its closest reference genome identified by StrainGST. To filter likely sequencing errors, bases with an Illumina Phred base quality score < 5 are ignored by default. An allele is considered strong if the sum of base quality scores supporting that allele is (i) higher than 50 (roughly equivalent to having at least two high-quality supporting reads) and (ii) at least 10% of the total sum of base quality scores of all alleles at that genomic position. If an allele is present but doesn’t match these criteria, it is considered weak. StrainGR stores weak evidence for use when tracking a strain across multiple samples—if a particular strain is highly abundant in some samples, with many strong SNP calls, then weak calls can be useful to discern that allele in low abundance samples from the same sample set.

Based on the observed alleles, StrainGR classifies a genomic position as either “reference confirmed,” “SNV” or “multiple alleles.” If a position has a single strong allele call, and that allele is the same as the reference, the position is classified as “reference confirmed.” A position with a single strong allele call that is different from the reference is classified as a SNV. Any position with multiple strong allele calls (whether they match the reference or not) is classified as “multiple alleles.”

To estimate the overall degree of similarity between the strain in the sample and its closest reference, StrainGR computes an estimate of average nucleotide identity (ANI) using StrainGR SNV calls: the average callable nucleotide identity (ACNI) is the percentage of positions marked as “reference confirmed” out of all positions with a single strong allele call.

##### Large deletion predictions

StrainGR also analyzes the read alignments to identify large deletions present in a specific strain within a sample, as compared to its closest reference identified by StrainGST. Consecutive positions in the reference genome over a specified length (by default 5000 bp; ~ 2 genes) with no aligned reads could indicate a large deletion. To account for situations with low coverage across the genome (< 1x), StrainGR employs a simple heuristic that exponentially scales the threshold for the length of such regions at lower coverages; thus, only longer gaps can be detected at lower coverages. If *λ* is the average depth of coverage along the genome, and *ϕ* is the unadjusted threshold, then the adjusted minimum size of a “gap” is:

$${\phi}^{\prime }=\frac{\phi }{1-\exp \left(-\lambda \right)}$$

Large deletions are used to assess whether particular genes are absent from the strain in a sample. In addition, the overall pattern of deletions across the genome for the strain in a longitudinal sample set can be used as a strain “fingerprint” to track a particular strain across samples.

##### Strain comparisons across samples

To assess whether the strains in two samples are the same (or very closely related), we compare both SNV calls (via pairwise ACNI) and patterns of large deletions. StrainGR calculates pairwise ACNI by dividing the number of positions where both samples have the same strong allele by the total number of positions where both samples have a single strong allele. To compare the pattern of predicted deletions between two samples, StrainGR calculates the Jaccard similarity: if *G*_1_ is the set of positions *not* marked as a large deletion in sample 1, and *G*_2_ is the set of positions *not* marked as a large deletion in sample 2, then the gap similarity *l* is defined as

$$l=\frac{\mid {G}_1\cap {G}_2\mid }{\mid {G}_1\cup {G}_2\mid }$$

Benchmarking StrainGE using simulated data and mock communities

#### Spiked metagenome generation

Unless otherwise noted, all synthetic metagenomes used for benchmarking were generated as follows: reads were simulated from the relevant genomes using ART [30] and merged with reads subsampled from a genuine metagenomic data set without detectable *E. coli* (accession SRS014613) as per MetaPhlan2 [31] and StrainGST*,* up to a fixed depth of 3 Gb. At this depth, strain coverages of 0.1x, 0.5x, 1x, and 10x corresponded to relative abundances of 0.016%, 0.083%, 0.16%, and 1.6%, respectively, assuming a 5 Mb *E. coli* genome.

#### StrainGST database for *Escherichia*

For construction of the *Escherichia* reference database, all complete *Escherichia* genomes available in NCBI RefSeq were downloaded in July 2019 (929 genomes total; Additional file 2). All tools required to construct the StrainGST database are included in the StrainGE suite (kmer counting, clustering, and database construction). The full database with 361 *Escherichia* genomes uses 7.3 Gb of disk space.

In order to set StrainGST’s default clustering threshold, we benchmarked its ability to correctly identify single strain and two-strain mixes using the metagenomic spike-in methods described below, using synthetic reads generated from 200 randomly selected *E. coli* genomes spiked into subsets of real metagenomic samples devoid of *E. coli*, to a total of 3 Gb. For the single-strain benchmarks, 200 samples were generated with 10x, 1x, 0.5x, and 0.1x coverage of each of the selected *E. coli* genomes (800 samples total). For the 2-strain mix benchmarks, 100 random 2-strain combinations from the set of 200 selected *E. coli* genomes were spiked in at each combination of 10x, 1x, 0.5x, and 0.1x coverage (10 coverage combinations, 1000 samples total). The 1800 benchmark cases were run using database clustering thresholds of 0.95, 0.90, 0.85, and 0.80 Jaccard k-mer similarity, corresponding to Mash distance ANIs of 99.89%, 99.77%, 99.63%, and 99.49%, respectively. For each threshold, we measured precision, recall, and F1 score for strain identification, with true positives being only those cases in which StrainGST identified the closest reference strain to the true strain as measured by Jaccard k-mer similarity. The clustering threshold of 0.90 generated the best combined results in each of the three metrics (Additional file 1: Table S5).

#### Phylogenetics and MLST typing of genomes in the *Escherichia* reference database

A single copy core (SCC) phylogeny was generated for the entire database of reference genomes. In brief, SynerClust [32] was used to generate clusters of orthologous genes (orthogroups). A concatenated alignment was generated for all single-copy, core orthogroups using MUSCLE [33]. A phylogenetic tree was constructed using FastTree v2.1.8 [34]. Phylogenetic trees were visualized using iTol [35].

MLST designations for each reference genome were predicted with the tool mlst (https://github.com/tseemann/mlst). Sequence types reported were based on the Achtman scheme. *E. coli* clade/phylogroup designation was determined using ClermonTyping (https://github.com/A-BN/ClermonTyping). For cases when there were missing or conflicting results between predicted typing and MASH groups, the clade designation for a given genome was selected based on where it was located in the SCC phylogeny with respect to unambiguous genome to clade designations.

#### Creation of four-strain *E. coli* mock community

Four phylogenetically distinct *E. coli* strains—H10407 (clade A), E24337A (clade B1), UTI89 (clade B2), and Sakai (clade E)—were cultured separately overnight at 37 °C in 2 mL of liquid LB media shaking at 200 rpm. The bacterial number in each culture was estimated via optical density and then combined at a ratio of 80% H10407, 15% UTI89, 4.9% Sakai, and 0.1% E24337A. Genomic DNA was then extracted from this mock community using the Qiagen MagAttract DNA Isolation Kit (Hilden, Germany), following manufacturer’s protocols. In two separate tubes, human genomic DNA was then added to the extracted *E. coli* DNA for final ratios of 99% human/1% *E. coli* (weight/weight). Sequencing data for this mock community has been submitted to NCBI’s Sequence Read Archive (SRA) under bioproject PRJNA685748 (biosample SAMN17091845).

#### Comparison of tools for tracking specific strains across samples using simulated sets of related samples

We compared the ability of StrainGE, StrainPhlan [15], and MIDAS [14] to track strains across samples. We performed strain tracking comparisons across ten sets of twelve spiked metagenomes, where each set of twelve was structured similarly in terms of strain content (Fig. 2a, b). For each set, we randomly picked two *Escherichia* reference genomes (A and B) from NCBI RefSeq complete and derived two different but closely related synthetic strains from each reference by introducing ~ 5000 random SNVs (99.9% ANI) uniformly across the genome. We spiked reads generated from these synthetic genomes into a real metagenome to generate samples containing these strains in different combinations (Fig. 2b), at 0.1x, 0.5x, 1x, and 10x coverage.

For each data set, we assessed strain similarity metrics calculated by each tool, to determine whether the tool could match (i) the identical strain found in different samples (i.e., strain A in sample 1 and 2; Fig. 2c), (ii) strains found either in mixtures or single-isolate samples (i.e., strain A in sample 1, 2, 5, and 6; Fig. 2d), or (iii) closely related strains (i.e., the ability to distinguish strain A1 from strain A2; Fig. 2e). In each case, we compared the tools’ predictions to the known strain content of each sample to calculate true positives (TP), false positives (FP), and false negatives (FN). For each tool, we varied the threshold (discussed in detail below) for determining shared strains in order to plot precision-recall curves.

##### Detecting shared strains using StrainGE

For each sample, we ran the complete StrainGE pipeline: StrainGST was run to identify the closest reference genomes, and StrainGR was run on a sample-specific concatenated reference to call genetic variation. To detect shared strains, we collected all samples predicted to match to the same StrainGST reference and computed a pairwise ACNI matrix for strain comparisons with at least a 0.5% callable genome. The similarity matrix was transformed to a distance matrix by computing 1 − *ACNI*, and transformed to a genetic distance using the Jukes-Cantor model [36]. If a pair of samples did not share any predicted close reference genomes, we set the distance between those samples to the maximum integer value. To plot the precision-recall curve, we varied the genetic distance threshold that determines when strains are considered the same.

##### Detecting shared strains with StrainPhlan

We ran StrainPhlan on each sample, using the tool’s marker gene database v295 (Jan 2019). Using the marker gene SNV profiles for each sample, StrainPhlan computed the pairwise sample distance matrix using Kimura’s two parameter model [37] (as suggested in their user manual). To plot the precision-recall curve, we varied the genetic distance threshold that determines when samples share a strain, as performed for StrainGE. To tune StrainPhlan for lower coverage levels, we ran it using --relaxed-parameters.

##### Detecting shared strains with MIDAS

We ran MIDAS v1.3.2 (database version v1.2) with default parameters. MIDAS includes a strain tracking tool that is first “trained” by giving it a single sample from each patient in a cohort. This training step identifies unique SNV markers for each patient. For our benchmarking, we “trained” MIDAS on samples containing a single strain (sample 1 for strain A1, sample 3 for strain B1, sample 7 for strain A2, and sample 9 for strain A2). (This likely helped the tool in benchmarking since, in a real world scenario, it is likely unknown whether a training sample contains a single strain.) Next, MIDAS compares these SNV markers to alleles in other samples and assesses how much they overlap. To plot precision-recall curves, we varied the percentage of overlapping markers between two samples that serves as a threshold to determine whether two samples share a strain. To tune MIDAS for lower coverage levels we ran its merge_snvs.py script with --all_snvs --all_samples and its strain_tracking.py script with --min_reads 1.

#### Evaluating the ability of StrainGR to quantify strain sharing in distinct metagenomic backgrounds

In order to determine how well StrainGE metrics recapitulated genetic relationships between strains, we generated another set of spiked metagenomic samples, spiked with varying quantities of *E. coli* reads from real, previously sequenced isolates. Ten stool metagenomes were randomly selected from the Human Microbiome Project [5] (Additional file 1: Table S3). The randomly selected samples contained *E. coli* at relative abundances between 0.005% and 0.9%; no two samples contained the same *E. coli* strain based on StrainGST output. Ten complete genome sequences of *E. coli* isolates, distinct from those identified in the background metagenomes, were selected from NCBI RefSeq database. For each isolate, ten variants were created by generating random mutations, such that the ANI to the original reference ranged from 99.9% to 99.99% at increments of 0.01%. Each reference and variant (110 in total) were spiked into at least two randomly chosen distinct metagenomic backgrounds at coverage levels of 0.1x, 0.5x, 1x, 2x, or 5x. A total of 300 synthetic samples were generated, with 350 pairs containing an identical strain in a distinct background. All spiked samples were analyzed with StrainGE; all sample pairs with a matching StrainGST reference were compared using StrainGR. StrainGST hits corresponding to strains present in background samples were not considered further. The ACNI was calculated for every pair.

### Evaluation of StrainGE on longitudinal, clinical metagenomic samples

#### Metagenomic time series from a patient with Crohn’s disease

We downloaded from the UCSD Qiita database (https://qiita.ucsd.edu/; Additional file 3) 27 metagenomic data sets representing stool longitudinally collected from a single individual with Crohn’s Disease [25]. We ran the full StrainGE pipeline on each sample, using our *Escherichia* database and default parameters, to identify and analyze *E. coli* strains. For StrainGR, to ensure each genome had sufficient unique content, we constructed a concatenated reference using StrainGE’s builtin prepare-ref tool, which performed another round of clustering of the StrainGST reported references at a default threshold of 99.2% ANI. The resulting reference contained 10 out of 14 total reported references (Fig. 4b; phylogroup G, B2 and A). For pairwise strain comparisons, we only included samples where the common callable percentage of the genome was > 0.5%.

#### Metagenomic sequencing of longitudinally collected stool

Twelve longitudinally collected stool samples were extracted with Chemagen Kit CMG-1091 (Baesweiler, Germany). Libraries were generated with NexteraXT (Illumina, San Diego, CA, USA) and sequenced in paired-end mode on an Illumina HiSeq 2500 (101 bp length) and/or Illumina HiSeq X10 (151 bp length). Short-read sequencing data was submitted to the Sequence Read Archive (SRA) with Bioproject accession PRJNA400628 (Additional file 3). We ran the full StrainGE pipeline on each sample, using our *Escherichia* database and default parameters, to identify and analyze *E. coli* strains. For pairwise strain comparisons, we only included samples where the common callable percentage of the genome was > 0.5%.

#### Characterization of *Enterococcus* strain diversity across a large cohort of babies

We downloaded data from by Shao et al. [27] from ENA, including 1679 metagenomes (accession ERP115334) and all isolate samples tagged as *Enterococcus* (accession ERP024601). We ran StrainGE on each metagenomic sample, using our *Enterococcus* database. To compare StrainGE’s ACNI to true ANI between the corresponding isolate genomes, we ran StrainGST on the raw isolate reads to identify a close reference genome, aligned the isolate reads to this reference using BWA-MEM [29], and used Pilon [38] to call variants. To compute the ANI between each pair of isolates that matched the same reference, we compared reference and alternative alleles called by Pilon where both samples had a single base call. For pairwise strain comparisons using StrainGR in the corresponding metagenomic samples, we only included pairs with a common callable genome > 0.5%.

## Supplementary Information

**Additional file 1.** Supplementary Tables, Figures and Text.

**Additional file 2.** Identifiers of genomes in the Escherichia and Enterococcus StrainGST reference databases.

**Additional file 3.** Identifiers of clinical gut microbiome datasets.

**Additional file 4.** Review history.

## Data Availability

The data generated and analyzed for this publication is available in SRA, under BioProject accession PRJNA400628 (patient with recurrent UTI), and PRJNA685748 (four *E. coli* strains mock community). The data from an individual with Crohn’s disease was previously published by Fang et al. [25] and available on QIITA (Artifact IDs 54476 and 54537). The data analyzed to investigate *Enterococcus* strain diversity in the gut microbiomes of babies was previously published by Shao et al. [27] and available from ENA (ERP115334 and ERP024601). StrainGE is an open source tool written in Python and C++ released under the BSD 3-clause license, available at https://github.com/broadinstitute/strainge [39]. StrainGE is built on top of several existing Python libraries, including NumPy [40] and SciPy [41]. Analysis scripts to generate the figures in this publication are available at https://github.com/broadinstitute/strainge-paper [42].
