# Supplementary material for: StrainGE: a toolkit to track and characterize low-abundance strains in complex microbial communities
Source: Genome Biol. 2022 Mar 7;23:74. doi: 10.1186/s13059-022-02630-0 (PMC8900328; doi:10.1186/s13059-022-02630-0)
Supplement: Supplementary file 4 — Additional file 4. Review history. [file 13059_2022_2630_MOESM4_ESM.docx]

**1^st^ round**

**Reviewer 1**

The article by van Dijk et al, describes two pieces of software to obtain strain level resolution of a metagenomic population. There is really no new idea in this paper; the software works by finding unique kmers among a collection of reference genomes and then competitively query the metagenomic reads against this unique kmer collection and provides means to visualize and further process the results. For instance, GOTCHA and Kraken use a similar strategy. Further, StrainGE analysis is limited when there is a mixture of novel strains in the metagenome or hybrid genomes of available strains (e.g. due to extensive recombination), similar to other existing tools and approach. That said, I believe, the capabilities provided by the two pieces of software, especially in terms of results processing, would be useful to the non-experts/non-bioinformaticians, who do not have the coding skills to do these tasks on their own. Hence, this seems to be a potentially useful contribution to the field. There are no new biological or evolutionary insights emerging form this study; the paper merely describes the tool and its applications with showcase examples from fecal/gut microbiomes.

The paper is well written overall but it is way too long and several of the results are repetitive. The authors should decide which are the results of secondary importance and move them to a supplementary material. I believe the paper could be reduced to almost half of it current length, without losing much in clarity or completeness. I have some more minor comments that require the attention of the authors in order to further improve the clarity of the manuscript.

I believe the authors should briefly discuss, up front, what strain definition is used in their analysis and also how many kmers should be detected for robust detection/presence. In the Discussion, I read that the most common definition used for strain used was 99.8% ANI and the authors suggest to adjust this definition depending on the specific needs of the project, which sounds OK to me, but, I believe, this information needs to come up much earlier, in the beginning of the Results in order for the readers to be able to follow better.

Line 10. Up to 1/3 different gene content is what has been usually seen for complete genomes, not more than half…

I felt that the niche that this tool fulfills was not described clearly in the Introduction section and thus, the justification for the study is not strong. I think the justification is that tools employing SNP patterns require typically higher coverage than tools employing kmer matching in general, and available kmer matching programs like GOTCHA and Kraken do not offer the processing capabilities of the data that StrainGE does, especially with respect to strain (as opposed to species) detection. These -and additional justification, if any- should be made clearer in the Introduction section, in the view of this reviewer.

Lines 34-43, page 6. Did the other tools have the same genomes as databases like StrainGE for these comparisons? If not, this is not a fair comparison (e.g., to MIDAS), and this was not made clear.

Figure 3. I thinκ it makes sense that callable ANI is lower than real/reference ANI because it does not include the conserved parts of the genomes (with identical kmer). This is ok, i believe. But I am not sure why kmer-based callable ANI is higher than isolate-based ANI in Figure 6 and I don't think the explanations provided by the authors explain this fully (e.g., plasmids considered in one calculation vs. the other, although it is true that plasmid sequences tend to be a bit less conserved than the chromosomal sequences, in general). I suspect that the difference has to do with the kmer-based approach not being optimized for a greater range of ANI values, meaning more divergent genomes than 99.5% ANI (note the differences in the scales of the two figures). This issue should be examined in more detail by the authors, I believe.

Line 13-21, page 12. What is the evidence that these strains truly exist in the sample? Without this information is not possible to fully evaluate the results of strainGE. Also, at what level of ANI strains are defined or unique? Not sure how to interpret the number of strains found without a definition of what a strain is, even approximate (later on, on page 19, I read about a 99.8% definition, but this needs to come up earlier and be clearly stated). Related to this, how many kmers per genome should there be as a minimum to detect the genome/strain? Some earlier studies showed that 10% of the genome is enough for adequate detection; what is used here and why?

**Reviewer 2**

In this manuscript, the authors present StrainGE a toolkit developed to study conspecific strains in microbial communities, with particular attention to low-abundance strains.

StrainGE consists of two main components allowing to recover the most similar strain references from a pre-built database and to define the relative abundances of the detected strains (StrainGST), and to precisely call SNVs with respect to such references (StrainGR).

Short-read based tools aiming at characterizing conspecific strains either work with sufficiently high depth of coverage or target specific genomic regions (e.g., single-copy marker gene sets). In this setting, StrainGE might provide a useful and complementary toolkit to study complex microbial environments, especially those concerning well studied species for which a large reference dataset could be built (e.g., Escherichia coli).

The manuscript is well written and, in my opinion, provides a quite thorough study of the presented method with syntethic and real datasets.

Nevertheless, I think there are still a couple of questions that should be addressed before I can fully recommend the manuscript for publication.

In the following, page number is relative to the actual manuscript.

Major remarks:

1) Computational requirements are completely missing.

CPU time and RAM of StrainGST and StrainGR should be compared against the tools chosen for comparison.

As the study involves a database construction, I think that also disk usage should be discussed in the manuscript.

2) The authors discarded some tools due to their focus on marker genes, yet also StrainGE limits its analysis on what it has been defined as "callable region".

Also, the authors claim at page 4 (lines 17-18) that "StrainGR provides additional detailed information about gene content and SNVs in a strain" in line 17-18 at page 4. As genes are mentioned, I think it could be informative to compare StrainGE against tools that characterize conspecific strains in single-copy-gene regions (e.g., DESMAN), by restricting the analysis/comparison on these specific regions.

3) Page 6 (last paragraph) & Table 1

I believe the abundance estimation should be included for each of the tools employed for the comparison, in order to provide a more precise comparison.

This information could be also added in Table 1 for each "called" strain. If neither StrainEst nor BIB provide such a metric, a tool such as DiTASiC could be introduced to the comparison (if I am not mistaken this tool should be able to do that).

Moreover, the ANI and abundance with respect to the closest database reference for the false positives (orange) could be added in Table 1?

Minor remarks:

- I think that some more tools should be cited in the Background. More precisely, I would consider citing STRONG (doi: 10.1186/s13059-021-02419-7) which is a very recent improvement over the already-cited DESMAN, ConStrains (doi: 10.1038/nbt.3319) which has been specifically developed to untangle conspecific strains, and DiTASiC (doi: 10.1093/bioinformatics/btx237) which is supposed to provide abundance estimation at the strain level.

- Page 4: although this first Result sub-section is supposed to briefly present the method, if the authors also agree, I would briefly mention also here the three scoring metics employed to select the references in StrainGST.

- Supplementary Figure 1: why an analysis with 4 uneven-abundance strains was not included? (e.g., 10x:1x:0.5x:0.1x)

- (optional) As a supplementary material, it could be interesting to report (maybe as part of Suppl.Results A?) a boxplot showing strain divergence in StrainGST database (and also at different database-size levels).

- Figure 2: could be worth to consider DiTASiC or ConStrains as a better tool with respect to StrainPhlan in the comparison?

- In my opinion, Discussion should be shortened a bit and made "lighter".

- Page 20, line 15: I might be mistaken but it seems the 0.02% is lower than the value reported earlier in the manuscript.

- Page 25, line 12: (just a comment) MAPQ value could be a metric to identify unique matches when XA field is not available.

- Page 26, "StrainGST database for Escherichia": it seems to me that there are also 3- and 4-strain benchmarks that should be taken into account in this section.

Grammar/style:

- Page 5, line 57-58: less well-studied -> "less studied"

- Figure 1b: typo "insuffiecient" -> "insufficient"

- (optional) Page 6, line 10: "929 at time of download" -> "929 in Month Year"

- Page 10: "Ninety two percent" -> "92%"

- Page 19, line 18: "determine a bacterial species' interactions" -> remove "a"

- Page 24, lines 36-37: "to called SNVs" -> "to call SNVs"

- Page 25, line 5: "BWA mem" -> "BWA-MEM"

- Page 26, line 30: "RefSEq" -> "RefSeq"

**Authors’ response**

**We thank the editor and reviewers for their time and constructive feedback. The points raised were of great help in streamlining and clarifying our main findings. In short, we have substantially shortened and streamlined the results and discussion, added a comparison of predicted relative abundances, included a comparison of computational requirements, and improved our ACNI to ANI comparison. Our response to the reviewers is in bold text throughout.**

**Reviewer #1:** The article by van Dijk et al, describes two pieces of software to obtain strain level resolution of a metagenomic population. There is really no new idea in this paper; the software works by finding unique kmers among a collection of reference genomes and then competitively query the metagenomic reads against this unique kmer collection and provides means to visualize and further process the results. For instance, GOTCHA and Kraken use a similar strategy. Further, StrainGE analysis is limited when there is a mixture of novel strains in the metagenome or hybrid genomes of available strains (e.g. due to extensive recombination), similar to other existing tools and approach. That said, I believe, the capabilities provided by the two pieces of software, especially in terms of results processing, would be useful to the non-experts/non-bioinformaticians, who do not have the coding skills to do these tasks on their own. Hence, this seems to be a potentially useful contribution to the field. There are no new biological or evolutionary insights emerging form this study; the paper merely describes the tool and its applications with showcase examples from fecal/gut microbiomes.

**We thank the reviewer for their time and comments. While we agree that the StrainGE algorithm builds on some previously published ideas, it represents, to our knowledge, a substantial advance over these earlier methods. Unlike these previous tools, StrainGE was specifically designed to excel at disentangling strain identities for low-abundance members of a complex metagenomic community (below 0.1%). For example, compared to reference-based tools (including GOTTCHA, Kraken, StrainEst and BIB), StrainGE provides much more detail for low-abundance strains, including tracking specific SNPs and indels. We have added text to the manuscript to clarify the unique features of StrainGE compared to earlier tools (lines 55-72).**

**While we do not describe results that point to fundamentally new biological or evolutionary insights (which we would be less inclined to describe in a Methods focused manuscript), we used three real datasets that we thought showcase the kinds of novel biological information that can be obtained using StrainGE, that could not have been obtained using earlier tools. In particular, StrainGE provided a more complete view of low-abundance strain content:**

1) **When examining a set of stool samples from a patient with Crohn’s disease, we were able to identify low abundance (<1%) and secondary strains that were not identified in a previous analysis using MIDAS, including evidence that specific strains persisted in the gut longer than previously noted.**
2) **In an analysis of longitudinal stool samples from a woman with a recurrent urinary tract infection, we were able to track a single *E. coli* strain that persisted over 10 months, despite its average abundance being only 0.6%, and despite it sometimes being found as part of a mixture of *E. coli* strains. We were able to track specific SNPs and indels across time within this strain.**
3) **In an analysis of *Enterococcus* in mother-child pairs using metagenomic information only, we were able to obtain results that previously could only be obtained using isolates.**

**As shown in these three examples, StrainGE was able to transform analysis of low-abundance species within metagenomic samples, allowing for accurate tracking of strains across time and between individuals, and yielding additional detailed information on shared SNPs and indels. We have added additional text in the discussion to make this point clear (lines 351-360).**

The paper is well written overall but it is way too long and several of the results are repetitive. The authors should decide which are the results of secondary importance and move them to a supplementary material. I believe the paper could be reduced to almost half of it current length, without losing much in clarity or completeness.

**We agree with the reviewer, and we have shortened the paper by approximately 25%. This included reorganizing the Results to streamline the benchmarking (lines 153-232), and substantially shortening the discussion, by approximately 50% (lines 337-395).**

I have some more minor comments that require the attention of the authors in order to further improve the clarity of the manuscript.

I believe the authors should briefly discuss, up front, what strain definition is used in their analysis and also how many kmers should be detected for robust detection/presence. In the Discussion, I read that the most common definition used for strain used was 99.8% ANI and the authors suggest to adjust this definition depending on the specific needs of the project, which sounds OK to me, but, I believe, this information needs to come up much earlier, in the beginning of the Results in order for the readers to be able to follow better.

**We thank the reviewer for pointing out the need for clarification. We have added text near the beginning of the Results to clarify our definition of a strain, as well as our thresholds (lines 131-134, 180-185).**

Line 10. Up to 1/3 different gene content is what has been usually seen for complete genomes,not more than half…

**We thank the reviewer for raising this point since it gives us an opportunity to clarify our meaning, which we have done by rewording lines 44-45 and adding a citation. About half of the genes in a typical *E. coli* genome are present in the “core” genome found in 99% of all *E. coli* [Touchon *et al.* 2020, Figure 1b]; the other half of genes are auxiliary and only shared with a subset of *E. coli*.**

I felt that the niche that this tool fulfills was not described clearly in the Introduction section and thus, the justification for the study is not strong. I think the justification is that tools employing SNP patterns require typically higher coverage than tools employing kmer matching in general, and available kmer matching programs like GOTCHA and Kraken do not offer the processing capabilities of the data that StrainGE does, especially with respect to strain (as opposed to species) detection. These -and additional justification, if any- should be made clearer in the Introduction section, in the view of this reviewer.

**We appreciate this feedback. We have now strengthened the justification for StrainGE in the introduction, including clarifying how the strengths of StrainGE compared to both reference based tools, including GOTTCHA and Kraken2, and SNP-based tools, such as StrainPhlan and MIDAS (lines 55-72).**

Lines 34-43, page 6. Did the other tools have the same genomes as databases like StrainGE for these comparisons? If not, this is not a fair comparison (e.g., to MIDAS), and this was not made clear.

**Wherever possible, we used the same databases for comparisons between tools. Of the tools that select close reference genomes from a database (StrainGST, StrainEst, BIB), we only used a different database for BIB, as it required a more computationally intensive database construction method that did not scale well. As a result, the database for BIB contained only 20 genomes. This is detailed in Supplementary Results B, and we have now added an explanation for this in the main text as well (lines 158-160).**

**The issue of database size did not apply to MIDAS or StrainPhlan, as these tools use SNVs to track strains across multiple samples and did not use a reference database.**

Figure 3. I thinκ it makes sense that callable ANI is lower than real/reference ANI because it does not include the conserved parts of the genomes (with identical kmer). This is ok, i believe. But I am not sure why kmer-based callable ANI is higher than isolate-based ANI in Figure 6 and I don't think the explanations provided by the authors explain this fully (e.g., plasmids considered in one calculation vs. the other, although it is true that plasmid sequences tend to be a bit less conserved than the chromosomal sequences, in general). I suspect that the difference has to do with the kmer-based approach not being optimized for a greater range of ANI values, meaning more divergent genomes than 99.5% ANI (note the differences in the scales of the two figures). This issue should be examined in more detail by the authors, I believe.

**We thank the reviewer for raising this issue, which we also pondered but initially decided not to dig into. We have now investigated further and determined that FastANI sometimes reports an artificially low ANI because two matched 3kb blocks on the query and reference can sometimes contain the boundary of a structural event in one member of the pair, *e.g.,* a large indel. As such, we opted to revise our analysis to include the variant caller Pilon (Walker et al., 2014), which we used, for each isolate data set, to identify SNVs against its closest StrainGST reference and then compared SNVs between isolates having the same StrainGST reference to calculate ANI. We then compared this value to StrainGR’s SNV-based ACNI computed between the metagenomes from which the isolates were derived, which revealed a much stronger and cleaner correlation than FastANI (Figure 6b). We have edited the Methods (lines 707-711) and the Results (lines 322-323) accordingly.**

Line 13-21, page 12. What is the evidence that these strains truly exist in the sample? Without this information is not possible to fully evaluate the results of strainGE.

**We appreciate this reviewer’s point, but believe that the predictions made were reasonable and likely to be correct given our benchmarking and consistency of the predictions made for some low abundance strains found in multiple samples. We benchmarked StrainGE extensively, including using a mock community of known composition that was created in the laboratory and then sequenced. We show that, for strains having relative abundances ranging from 0.02-1.6% (or 0.1x-10x), we could accurately pinpoint the correct reference strain in all cases. This gave us confidence in the strains predicted from the Fang *et al.* dataset. The average depth of coverage along reference *E. coli* EC-1369 varies between 1-2x in time points 6, 7 and 9, which is higher than what we used in most benchmarks. In addition, the low abundance strains that we identified (i.e. light green strains identified in timepoints 7 and 9) are almost identical to each other (ACNI 99.98%), which would be unlikely if the predictions were based on noise alone. Although we do not have isolates cultured from these low-abundance timepoints, our results are consistent with persistence of *E. coli* across time points within the gut, a known feature of *E. coli* in the gut.**

Also, at what level of ANI strains are defined or unique? Not sure how to interpret the number of strains found without a definition of what a strain is, even approximate (later on, on page 19, I read about a 99.8% definition, but this needs to come up earlier and be clearly stated).

**To clarify the definition of a unique strain, we have added additional text into the beginning of the Results (lines 131-134). While the minimum distance between references in our StrainGST database is indeed 99.8% ANI, StrainGR allows us to further refine relationships among strains that map to the same reference. In this manuscript, when comparing strains in different samples matching the same reference with StrainGR, we used a stricter threshold of 99.95% ACNI to define a strain. However, StrainGE allows the user flexibility to tailor the definition of a strain to a particular study.**

Related to this, how many kmers per genome should there be as a minimum to detect the genome/strain? Some earlier studies showed that 10% of the genome is enough for adequate detection; what is used here and why?

**StrainGST balances multiple factors to call a strain as present, and thus doesn’t have a defined minimum threshold specifically for kmers per genome. The factors included in this combined metric are: i) the fraction of reference k-mers that are present in the sample; ii) how evenly spaced across the genome the k-mers are (as reflected in our evenness score); and iii) the fraction of the k-mer counts in the sample that are explained. A threshold resulting from this final combined score is used to call a strain as present, and this threshold was optimized using benchmarks (Supplementary Results A).**

**Few k-mers are required for StrainGST to correctly report a reference genome because StrainGST uses genome sketches, similar to Mash (Ondov *et al., 2016*), sized to 1% of the total number of k-mers in a genome (lines 408-409). Furthermore, not all k-mers in a genome sketch need to be present. For the analysis of the *in vitro* mock community (Table 1), StrainGST considered 1% of the kmers in the most abundant strain present in the mixture (*E. coli* SEC470; all k-mers in genome sketch were present), and only 0.35% of the k-mers in the least abundant strain (*E. coli* 24377A). Even using such low fractions of the k-mers in the reference genome, StrainGST reported the correct reference genomes in our benchmarking.**

**Reviewer #2:** In this manuscript, the authors present StrainGE a toolkit developed to study conspecific strains in microbial communities, with particular attention to low-abundance strains.

StrainGE consists of two main components allowing to recover the most similar strain references from a pre-built database and to define the relative abundances of the detected strains (StrainGST), and to precisely call SNVs with respect to such references (StrainGR).

Short-read based tools aiming at characterizing conspecific strains either work with sufficiently high depth of coverage or target specific genomic regions (e.g., single-copy marker gene sets). In this setting, StrainGE might provide a useful and complementary toolkit to study complex microbial environments, especially those concerning well studied species for which a large reference dataset could be built (e.g., Escherichia coli).

The manuscript is well written and, in my opinion, provides a quite thorough study of the presented method with syntethic and real datasets.

**We thank the reviewer for their time and kind feedback.**

Nevertheless, I think there are still a couple of questions that should be addressed before I can fully recommend the manuscript for publication.

In the following, page number is relative to the actual manuscript.

Major remarks:

1) Computational requirements are completely missing.

CPU time and RAM of StrainGST and StrainGR should be compared against the tools chosen for comparison.

As the study involves a database construction, I think that also disk usage should be discussed in the manuscript.

**Thank you for pointing out this omission. We have added a comparison of computational requirements in Supplemental Figure 9, and supporting text in the results (lines 226-228, 585-586). This new analysis shows that the runtime of StrainGE is comparable to that of StrainPhlan and MIDAS, and memory usage is well within the range readily available on modern cluster systems or powerful laptops.**

2) The authors discarded some tools due to their focus on marker genes, yet also StrainGE limits its analysis on what it has been defined as "callable region".

**StrainGE’s “callable” regions maximize the information that can be obtained from strain-specific regions for a given dataset; marker genes would represent a subset of this. For example, in our strain tracking benchmark comparing StrainGE to StrainPhlan and MIDAS, StrainGE’s mean callable genome is 74% ± 13% (at 10x coverage), while StrainPhlan’s marker genes only cover on average 1.4% ± 0.3% of the relevant *E. coli* references. We have added this comparison to the manuscript (lines 223-225, 327-328).**

**However, we did not exclude tools purely for using marker genes, as we included StrainPhlan in our comparisons. We did exclude ConStrains, another tool which uses marker genes, due to its hard requirement for a minimum coverage of ≥10x (Luo *et al.* 2015), which was confirmed independently in another study (Quince *et al.* 2017). We have clarified our rationale for excluding ConStrains in the manuscript (lines 202-203).**

Also, the authors claim at page 4 (lines 17-18) that "StrainGR provides additional detailed information about gene content and SNVs in a strain" in line 17-18 at page 4. As genes are mentioned, I think it could be informative to compare StrainGE against tools that characterize conspecific strains in single-copy-gene regions (e.g., DESMAN), by restricting the analysis/comparison on these specific regions.

**DESMAN is not designed to operate at the low coverages that we are examining in this manuscript. Since it requires *de novo* metagenomic assembly, it is unlikely to produce usable contigs at the coverage levels for which StrainGE was designed to operate. However, as single-copy-core regions are less likely to be part of callable regions, we do not expect StrainGR to be as informative within single-copy-core regions.**

3) Page 6 (last paragraph) & Table 1

I believe the abundance estimation should be included for each of the tools employed for the comparison, in order to provide a more precise comparison.

This information could be also added in Table 1 for each "called" strain. If neither StrainEst nor BIB provide such a metric, a tool such as DiTASiC could be introduced to the comparison (if I am not mistaken this tool should be able to do that).

**We thank the reviewer for raising this point. Unlike StrainGE, StrainEst and BIB only give relative abundances within the species of interest, not as a percentage of the whole sample. For completeness, we now include these values in Supplemental Table 3.**

**We have attempted to run DiTASiC as an alternative; however, we had problems getting the program to run to completion since it halts if fewer than 75% of reads can be assigned to a reference in the database. As *E. coli* was present at a relative abundance of at most 1.6%, DiTASiC did not complete using our *Escherichia*-specific database. Even when including references for additional non-*E. coli* species detected by MetaPhlan in our sample, we were not able to get DiTASiC to align the requisite 75% of reads. We expect that DiTASiC works well in some scenarios but this led us to conclude that it would not be the preferred approach for strain investigations of low abundance species that is enabled by StrainGE. Although we have not included DiTASiC in our comparisons, we have added discussion of DiTASiC and its citation to the introduction (lines 58-59).**

Moreover, the ANI and abundance with respect to the closest database reference for the false positives (orange) could be added in Table 1?

**Thank you for this suggestion. We have added the ANI to the closest true strain for each of the reported false positives into Table 1 (p. 8-9).**

Minor remarks:

- I think that some more tools should be cited in the Background. More precisely, I would consider citing STRONG (doi: 10.1186/s13059-021-02419-7) which is a very recent improvement over the already-cited DESMAN, ConStrains (doi: 10.1038/nbt.3319) which has been specifically developed to untangle conspecific strains, and DiTASiC (doi: 10.1093/bioinformatics/btx237) which is supposed to provide abundance estimation at the strain level.

**We thank the reviewer for pointing out these omissions, and we have now included them in the introduction (lines 58-69).**

- Page 4: although this first Result sub-section is supposed to briefly present the method, if the authors also agree, I would briefly mention also here the three scoring metics employed to select the references in StrainGST.

**Thank you for raising this point. We have added the introduction of the three scoring metrics to the first Result sub-section (lines 104-107).**

- Supplementary Figure 1: why an analysis with 4 uneven-abundance strains was not included? (e.g., 10x:1x:0.5x:0.1x)

**We thank the reviewer for this question. We did indeed include a benchmarking of this kind using a laboratory created mock community (lines 164-169) including 4 *E. coli* strains mixed at uneven-abundance in an approximate abundance ratio of 80:15:4.9:0.1. When we tested StrainGST on the sequencing data generated from this sample, StrainGST was the only tool able to recover the identities of all four strains without false positives.**

**For our synthetic spike-in benchmarking, it is true that we did not include mixtures of more than three strains at uneven abundances, but attempted to make the three strain mixtures very challenging to disambiguate by varying the relative coverage and genetic relationships of strains in the mixture. For example, the 10:1x:0.5x samples had a 20-fold difference in coverage between the highest and lowest abundant strain. While it would be possible to include these additional benchmarks, given the relative performance of StrainGE compared to the other tools, demonstrations of StrainGE’s ability to identify strains in real world scenarios, and that we agree that shortening the length of our manuscript would be prudent, we have decided not to include this additional work, but would be happy to do so if desired.**

- (optional) As a supplementary material, it could be interesting to report (maybe as part of Suppl.Results A?) a boxplot showing strain divergence in StrainGST database (and also at different database-size levels).

- **We thank the reviewer for this suggestion. We have added a supplemental figure showing for each strain in each database the ANI to its closest neighbor in Supplemental Figure 2.**
- Figure 2: could be worth to consider DiTASiC or ConStrains as a better tool with respect to StrainPhlan in the comparison?

**We thank the reviewer for raising this point. As discussed above, neither of these tools were designed to operate at the very low coverages that we are considering (~1% relative abundance of *E. coli* in the human gut).**

**We tried running DiTASiC, but were unable to get it to run to completion due to its requirement for having >75% of the reads align to the reference dataset. ConStrains also required substantially higher coverage. We have clarified our justification for not benchmarking these tools in the manuscript (Results, lines 202-203; Supplemental Text, lines 110-112).**

- In my opinion, Discussion should be shortened a bit and made "lighter".

**We agree with the reviewer, and have now shortened the discussion by approximately 50%.**

- Page 20, line 15: I might be mistaken but it seems the 0.02% is lower than the value reported earlier in the manuscript.
- **We apologize for the confusion, and have now clarified the different contexts for these two values (lines 134-137). Whereas StrainGST is accurate at a coverage as low as 0.1x (or 0.02% relative abundance in a 3G sample), for StrainGR a higher coverage level (0.5x, or less than 0.1% relative abundance) substantially improves its ability to track strains across samples (Figure 3).**
- Page 25, line 12: (just a comment) MAPQ value could be a metric to identify unique matches when XA field is not available.

**We thank the reviewer for the suggestion; however, StrainGR utilizes the actual alternative alignment location and CIGAR string encoded in the XA tag to mark every possible alignment location as potentially “shared genome”.**

- Page 26, "StrainGST database for Escherichia": it seems to me that there are also 3- and 4-strain benchmarks that should be taken into account in this section.

**The threshold obtained from this benchmark also performed well in our main StrainGST comparison (Supplemental Results A) with samples containing 1-4 strains, as well as a our benchmarking of the *in vitro* mock community, containing 4 strains of uneven composition.**

Grammar/style:

- Page 5, line 57-58: less well-studied -> "less studied"
- Figure 1b: typo "insuffiecient" -> "insufficient"
- (optional) Page 6, line 10: "929 at time of download" -> "929 in Month Year"
- Page 10: "Ninety two percent" -> "92%"
- Page 19, line 18: "determine a bacterial species' interactions" -> remove "a"
- Page 24, lines 36-37: "to called SNVs" -> "to call SNVs"
- Page 25, line 5: "BWA mem" -> "BWA-MEM"
- Page 26, line 30: "RefSEq" -> "RefSeq"

**We have fixed these typographical errors.**

**2^nd^ round**

**Reviewer 1**

The revised article by Dijk et al represents a much-improved version of the original manuscript and the authors have addressed all my major concerns. I have only a few remaining, minor points.

Line 75 and elsewhere. It would be nice to say what minimum coverage will be required either as fraction of total kmer of the genome (breadth) or X times the genome is covered by reads in the abstract and introduction, I think. That said, Introduction section reads much better now.

Line 124-128. I think the authors should define what callable ANI is here. The previous sentence is not clear, even after I read it twice.

Line 133. Above text mentions 99.8% (for strain definition). Not a big difference obviously but please check in order to be consistent.

Line 133-135. (related to my comment above). This information could be summarized in the abstract since it is an important take-away message, in my view at least.

Line 423 and elsewhere. Some characters do not show up well on my pdf version of the article like here and elsewhere in the Methods section. Please check.

**Reviewer 2**

I am happy with the responses provided by the authors and I think the manuscript improved compared to the previous version.

I had some issues related to the display of some equations. I assumed they did not change compared to the previous manuscript.

I think that StrainGE toolkit could be of great interest for biologists interested in strain-level analyses.

I have just some really minor suggestions/typos to point out:

Line 55. "to resolve." -> these words can be deleted from the end of the sentence.

Line 132. "depends" -> "depend"

Line 173. "whether" -> "if"

Line 230. "output" -> "outputted"

Line 231. remove comma before "do not offer convenient..."

Line 302. "strain level diversity" -> either "strain-level diversity" or "strain diversity"

**Authors’ response**

> Reviewer #1: The revised article by Dijk et al represents a much-improved version of the original manuscript and the authors have addressed all my major concerns. I have only a few remaining, minor points.

> Line 75 and elsewhere. It would be nice to say what minimum coverage will be required either as fraction of total kmer of the genome (breadth) or X times the genome is covered by reads in the abstract and introduction, I think. That said, Introduction section reads much better now.

We thank the reviewer for their feedback and have now included the minimum coverage required for StrainGE in the Abstract, as well as in the Introduction.

> Line 124-128. I think the authors should define what callable ANI is here. The previous sentence is not clear, even after I read it twice.

We have clarified the definition of “common callable” positions used to calculate the average callable nucleotide identity (ACNI) in the text (see two paragraphs on page 4 starting with “To separate SNVs belonging to different strains, …”). We have also added more explanation of callable positions in the legend for Figure 1.

> Line 133. Above text mentions 99.8% (for strain definition). Not a big difference obviously but please check in order to be consistent.

We apologize for the confusion. There are two thresholds to consider: first, the default database clustering threshold is set to 99.8% ANI, and defines the granularity of the StrainGST reference database. However, two different samples with strains matching the same reference are not necessarily the same. Thus, we run StrainGR on both samples individually to obtain variant calls. To assess whether the strains are the same, we compare positions where StrainGR was able to make a “strong” reference or alternative allele call in both samples (which we define as the common callable genome). The percentage of common callable positions where both samples have a single identical call equals our average callable nucleotide identity (ACNI) metric. If ACNI is >99.95%, we consider two strains to be the same. We have further clarified this in the manuscript (added sentence “This threshold is stricter than our initial database clustering threshold of 99.8%, as samples matching the same reference in the database can contain different strains”).

> Line 133-135. (related to my comment above). This information could be summarized in the abstract since it is an important take-away message, in my view at least.

We thank the reviewer for this suggestion. While our benchmarking of E. coli led us to apply ACNI >99.95% as our same-strain definition, we are reluctant to codify this threshold as a universal definition of a strain since we anticipate that this number may be different for other microbes and/or the definition of a “strain” may be dependent upon the research question. Thus, we intentionally made the thresholds easily tunable and we guide the user on how to use StrainGE to optimize these thresholds.

> Line 423 and elsewhere. Some characters do not show up well on my pdf version of the article like here and elsewhere in the Methods section. Please check.

We thank the reviewer for their careful read of our manuscript, including equations. We have corrected all issues with the equations in the original Word document.

> Reviewer #2: I am happy with the responses provided by the authors and I think the manuscript improved compared to the previous version.

> I had some issues related to the display of some equations. I assumed they did not change compared to the previous manuscript.

> I think that StrainGE toolkit could be of great interest for biologists interested in strain-level analyses.

> I have just some really minor suggestions/typos to point out:

> Line 55. "to resolve." -> these words can be deleted from the end of the sentence.

> Line 132. "depends" -> "depend"

> Line 173. "whether" -> "if"

> Line 230. "output" -> "outputted"

> Line 231. remove comma before "do not offer convenient..."

> Line 302. "strain level diversity" -> either "strain-level diversity" or "strain diversity"

We thank the reviewer for their positive feedback and additional suggestions to help us further improve our manuscript. All suggested changes have been made.
